# Diversity, habitat endemicity and trophic ecology of the fauna of Loki’s Castle vent field on the Arctic Mid-Ocean Ridge

**DOI:** 10.1038/s41598-023-46434-z

**Published:** 2024-01-02

**Authors:** Mari Heggernes Eilertsen, Jon Anders Kongsrud, Anne Helene Solberg Tandberg, Tom Alvestad, Nataliya Budaeva, Luis Martell, Sofia P. Ramalho, Tone Falkenhaug, Rony Huys, Eivind Oug, Torkild Bakken, Tore Høisæter, Cessa Rauch, Francisca C. Carvalho, Alexandra S. Savchenko, Tone Ulvatn, Katrine Kongshavn, Cassandra Mari Berntsen, Bernt Rydland Olsen, Rolf Birger Pedersen

**Affiliations:** 1 Department of Biological Sciences, University of Bergen, Bergen, Norway; 2 Center for Deep Sea Research, University of Bergen, Bergen, Norway; 3 Department of Natural History, University Museum of Bergen, University of Bergen, Bergen, Norway; 4 Centre for Environmental and Marine Studies (CESAM) and Department of Biology, University of Aveiro, Aveiro, Portugal; 5 Institute of Marine Research, Flødevigen Research Station, His, Norway; 6 Department of Life Sciences, Natural History Museum, London, UK; 7 Norwegian Institute for Water Research, Region South, Grimstad, Norway; 8 Norwegian University of Science and Technology, NTNU University Museum, Trondheim, Norway; 9 Loddefjord, Norway; 10 Invertebrate Zoology Department, Biological Faculty, Moscow State University, Moscow, Russia; 11 Western Norway University of Applied Sciences, Bergen, Norway; 12 Department of Earth Sciences, University of Bergen, Bergen, Norway; 13 Lørenskog, Norway

**Keywords:** Marine biology, Stable isotope analysis, Taxonomy, Biodiversity

## Abstract

Loki’s Castle Vent Field (LCVF, 2300 m) was discovered in 2008 and represents the first black-smoker vent field discovered on the Arctic Mid-Ocean Ridge (AMOR). However, a comprehensive faunal inventory of the LCVF has not yet been published, hindering the inclusion of the Arctic in biogeographic analyses of vent fauna. There is an urgent need to understand the diversity, spatial distribution and ecosystem function of the biological communities along the AMOR, which will inform environmental impact assesments of future deep-sea mining activities in the region. Therefore, our aim with this paper is to provide a comprehensive inventory of the fauna at LCVF and present a first insight into the food web of the vent community. The fauna of LCVF has a high degree of novelty, with five new species previously described and another ten new species awaiting formal description. Most of the new species from LCVF are either hydrothermal vent specialists or have been reported from other chemosynthesis-based ecosystems. The highest taxon richness is found in the diffuse venting areas and may be promoted by the biogenic habitat generated by the foundation species *Sclerolinum contortum*. The isotopic signatures of the vent community of LCVF show a clear influence of chemosynthetic primary production on the foodweb. Considering the novel and specialised fauna documented in this paper, hydrothermal vents on the AMOR should be regarded as vulnerable marine ecosystems and protective measures must therefore be implemented, especially considering the potential threat from resource exploration and exploitation activities in the near future.

## Introduction

Loki’s Castle Vent Field (LCVF, ca. 2300 m) was discovered in 2008 as the first black-smoker vent field on the Arctic Mid-Ocean Ridge^1^. At hydrothermal vents, geothermally heated fluids with dissolved energy-rich chemical compounds are expelled from the seafloor, fueling ecosystems based on chemosynthetic primary production^2^. The organisms inhabiting hydrothermal vents are exposed to extreme and fluctuating environmental conditions, such as high temperatures, toxic compounds (H_2_S, heavy metals) and high or low pH^3^. In addition, many species inhabiting vents have dietary adaptations to acquire nutrients from chemosynthetic microorganisms, such as symbiosis or bacterial grazing^4^. The combination of environmental tolerance and dietary adaptations leads to a high degree of specialisation in vent fauna and a large proportion of it is either endemic to this habitat or shared with other chemosynthesis-based ecosystems such as cold seeps and organic falls^5^.

**Figure 1:**
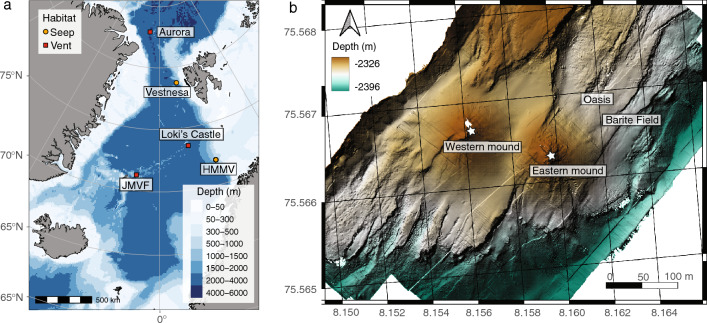
Maps showing the position (**a**) and layout (**b**) of Loki’s Castle vent field. Other Arctic vents and seeps mentioned in this paper are also illustrated (**a**). JMVF—Jan Mayen vent fields, HMMV—Håkon Mosby mud volcano. The two mounds at LCVF are indicated as western and eastern mound (**b**), with the main black smoker chimneys indicated by stars. The Barite Field and the Oasis are diffuse venting areas (**b**). The overview map (**a**) was generated in R^10^ using the ggOceanMaps package^11^, which utilised bathymetry from NOAA^12^ and land polygons from Natural Earth Data^13^. The bathymentric map of the LCVF (**b**) was generated using QGIS^14^ and GMT^15^.

The Arctic Mid-Ocean Ridge (AMOR) is an ultra-slow spreading ridge, with spreading rates between 20 mm/year at the Kolbeinsey Ridge north of Iceland, 15 mm/year on the Mohns Ridge and 10 mm/year at the Knipovich Ridge^6^. Loki’s Castle has two large mounds of hydrothermal sulphide deposits (Fig. 1), and preliminary analyses of plume fallout in hemipelagic sediments adjacent to the vent field suggest that hydrothermal activity commenced approximately 10,000 years ago. The black smoker fluids of the LCVF have a maximum temperature of 310–320 °C^1^, and the geochemical composition indicates thermal degradation of sedimentary organic matter below the seafloor^7^. However, the black-smoker chimneys are growing on a basaltic ridge, and thus the vent field is not sediment-covered^1,7^. Below the chimneys, the mounds are covered with chimney rubble, and there are areas with visible, shimmering fluids venting, so this whole area is considered active. Most of the vent fauna on the mounds is found near the base of the black smokers, on low-activity chimneys and on the chimney rubble. In addition to the mounds and black-smoker chimneys, the north-eastern flank of LCVF has an area of diffuse, low temperature venting of fluids that are formed by subseafloor mixing of at least 10% high-temperature hydrothermal fluids with cold (− 0.7 °C) seawater^8^. The diffuse venting is found in two adjacent areas called the Barite Field, named for the abundant barite chimneys^9^, and the Oasis, named for the high densities of tubeworms (Fig. 1). The major differences between the Barite Field and the Oasis are the lack of barite chimneys and the presence of pillow lava with diffuse venting through cracks in the latter area.

Prior to the discovery of LCVF, the only vent fields on the AMOR where the faunal community had been characterised were the shallower Jan Mayen Vent Fields (JMVF, 500–750 m depth), which are mainly inhabited by background fauna^16^. It is a common pattern globally that shallower hydrothermal vents have less specialised fauna, probably because of a higher influx of photosynthetically derived organic matter that reduces the evolutionary pressure leading to specialisations^17^. At LCVF, on the other hand, the fauna studied so far indicates the presence of a clearly specialised community^18–21^. The paper reporting on the discovery of Loki’s Castle included a brief description of the major faunal components of the vent field and speculated that the fauna had more in common with vent fields in the Pacific than those in the Atlantic^1^. Several new species have been described from LCVF, including the polychaetes *Nicomache lokii* (Maldanidae)^19^, *Pavelius smileyi* and *Paramytha schanderi* (Ampharetidae)^18^ and the amphipods *Exitomelita sigynae* (Melitidae)^20^ and *Monoculodes bousfieldi* (Oedicerotidae)^21^. However, a comprehensive faunal inventory of the LCVF has not yet been published, hindering the inclusion of the Arctic in biogeographic analyses of vent fauna. The fauna of hydrothermal vents globally forms distinct bioregions with a very high degree of endemicity (up to 95%) within each region^22^. Preliminary data from LCVF and the Aurora Vent Field on the Gakkel Ridge (3800 m)^23^, indicate that the Arctic vent fauna is very different from other ocean regions, but a more comprehensive dataset with high taxonomic resolution is needed to test whether the Arctic might form a distinct bioregion for vent fauna.

Deep-sea ecosystems along the Norwegian part of the AMOR may soon be facing disturbances from industrial exploration and exploitation relating to seabed mining. An opening process for mining of seafloor mineral resources on the AMOR was initiated in 2019, and the Norwegian government announced in June 2023 that they aim to open the suggested area for industrial exploration^24^. AMOR has a complex topography and hosts several vulnerable marine ecosystems such as seamounts with dense sponge aggregations^25^, sedimented areas with crinoid fields^26^ and hydrothermal vents^1,16^. Potential risks posed by seabed mining include habitat alteration or removal, sediment plumes from vehicles or mine tailings, which will extend to areas beyond the mined sites, and other vehicle-related disturbances such as light or noise pollution^27^. The dependence of the specialised vent fauna on a habitat that occurs only in small patches implies that vent ecosystems might be particularly vulnerable to anthropogenic impacts^28^. To assess the environmental impact of seabed mining activities in the AMOR region and enable spatial management plans, it is critical to understand the baseline biodiversity and spatial distribution patterns, as well as ecosystem functioning and food web structures in the region^29^.

Our aim with this paper is to provide an overview of the diversity, habitat endemism and trophic ecology of the vent fauna at LCVF and discuss links to other related habitats regionally and globally. This will be accomplished by compiling a comprehensive species inventory based on material collected on nine cruises since 2007, supported by a dataset linking the identifications to voucher material deposited at the University Museum of Bergen (Norway) and DNA barcodes. We also present a first insight into the foodweb of the LCVF based on stable isotope analyses.

## Results

### Faunal inventory

**Figure 2:**
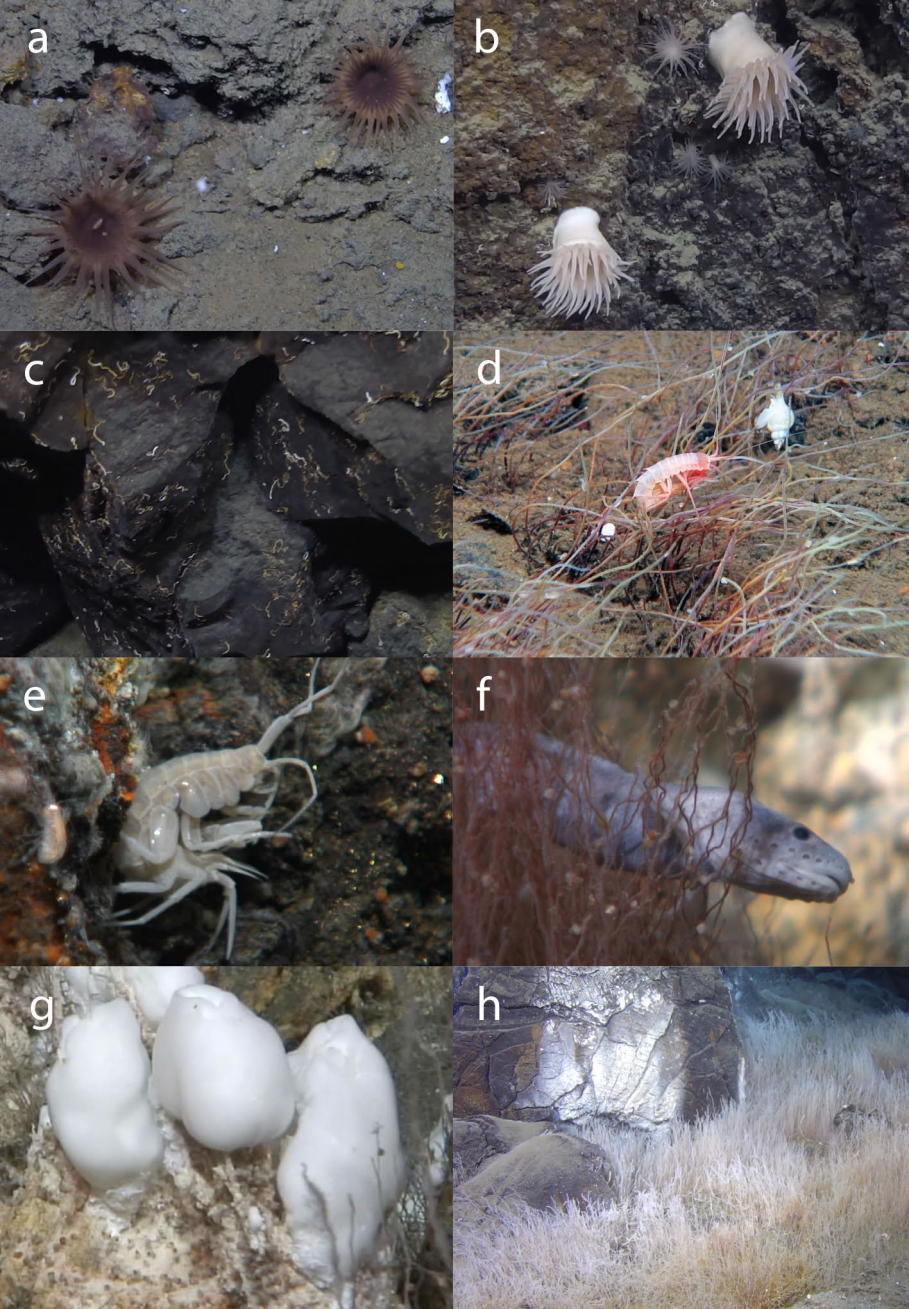
In situ images of fauna from the Loki’s Castle vent field. (**a**,**b**) Anemones (Actiniaria, several families) from the mounds, (**c**) Serpulidae from the Oasis, (**d**) *Sclerolinum contortum* tubes in the Barite Field with *Cleippides quadricuspis* and *Mohnia mohni*, (**e**) *Exitomelita sigynae* on the base of a chimney, (**f**) Zoarcidae indet. behind tubes of *S. contortum* in the Barite Field, (**g**) Large bacterial mats in the Barite Field with *Rissoa griegi* at the base, (**h**) Dense forests of *S. contortum* in the Oasis. Image credit: Centre for Deep Sea Research, University of Bergen.

A total of 62 taxa were recorded from the LCVF (Table 1). The most diverse phylum was Arthropoda with 22 taxa, of which 12 are amphipods. The second most diverse was Annelida with 18 taxa. The highest taxon richness is recorded from the diffuse-venting areas, especially the Barite Field which had 33 recorded taxa. The species which are vent specific or shared with other chemosynthesis-based ecosystems (CBEs) such as seeps or organic falls, and not recorded from outside these habitats, are considered specialised fauna (17 species in total). The specialised fauna at LCVF consisted of 11 annelids, 3 amphipods, 2 gastropods and one species of cyclopoid copepod. Some taxa that were identified only to higher taxonomic levels (e.g. nemerteans, halacarid mites, actiniarians) are quite abundant and might also include specialised species. The Actiniaria at LCVF belong to the families Actinostolidae, Hormathiidae, and Kadosactinidae, all of which are known for including taxa associated with deep sea habitats and hydrothermal vents^30–32^ (Fig. 2a,b). There are two eelpouts collected from the LCVF, and the most common of these was only identified to family level (Fig. 2f). Several zoarcid species are known to be vent-endemic^33^, and the high abundance of Zoarcidae indet. at LCVF and its close association with active areas in the vent field could indicate that this species is a vent specialist. Because of the unresolved taxonomic status of Zoarcidae indet., habitat endemism for this species was not assessed, but further morphological and phylogenetic analyses of this species are ongoing. A more detailed description of the taxonomic status and ecological remarks for each taxon group recorded can be found in Supplementary Notes.

**Table 1:**
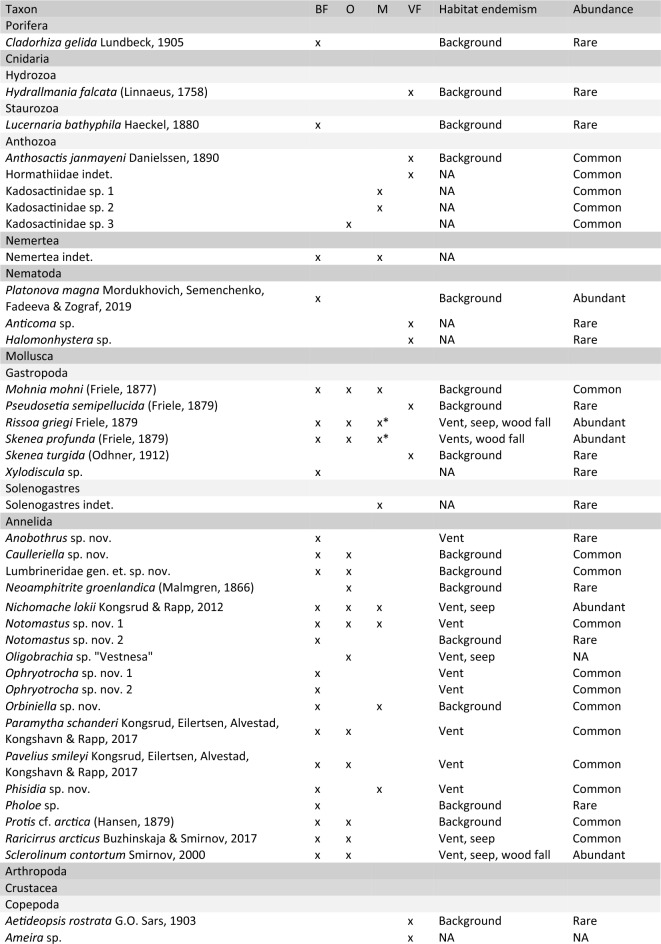

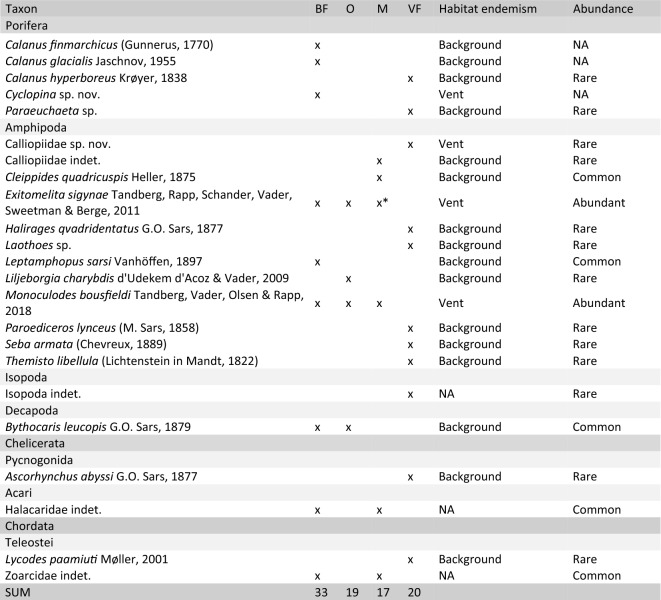
Faunal inventory for Loki’s Castle vent field.

The area each taxon has been collected from is indicated as BF (Barite Field), O (Oasis), M (Mounds, including chimneys) or VF (Vent Field) for samples where the exact sampling spot in the vent field is unknown. Habitat endemism is listed as Background if the species is known from non-CBE ecosystems, for CBE specialists the type of ecosystem is listed (Vent/Seep/Wood fall), and NA for not assessed. Abundance is semi-quantitative with categories reflecting the approximate number of collected specimens as follows: Rare = 0–10 specimens, Common = 10–100 specimens, Abundant =  > 100 specimens.

*Collected from near high temperature venting on the black smoker chimneys.

**Figure 3:**
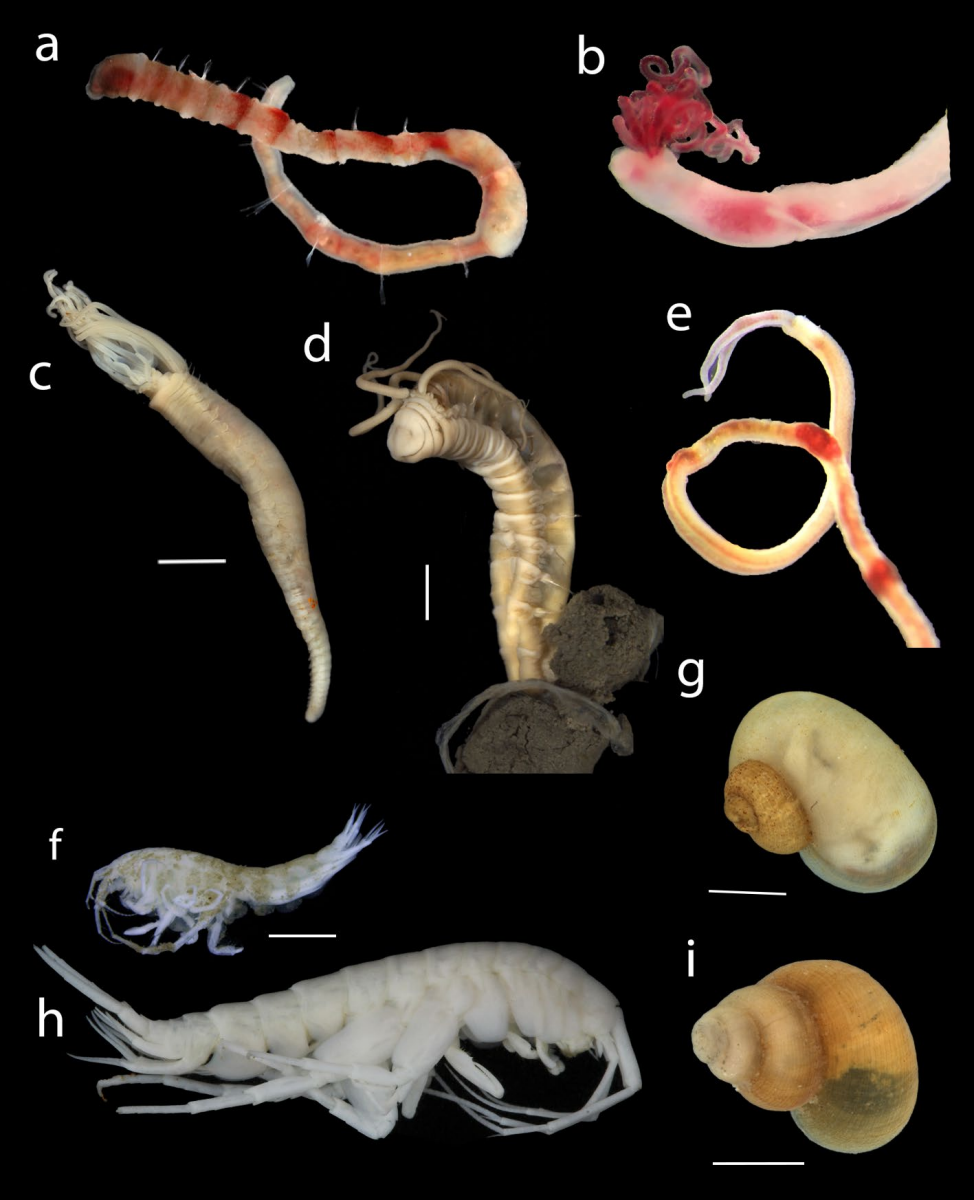
Images of fauna from LCVF. (**a**) *Nicomache lokii* (anterior part), (**b**) *Oligobrachia* sp. “Vestnesa” (anterior part), (**c**) *Paramytha schanderi,* (**d**) *Pavelius smileyi*, (**e**) *Sclerolinum contortum* (anterior part), (**f**) *Monoculodes bousfieldi*, (**g**) *Skenea profunda*, (**h**) *Exitomelita sigynae*, (**i**) *Rissoa griegi*. Scalebars: (**c**,**d**,**f**) 2 mm, (**g**,**i**) 1 mm. Image credit: Department of Natural History, University Museum of Bergen (**a**, **f**–**i**), Kongsrud et al. (2017) (**c**,**d**), N Rimskaya-Korsakova (**b**,**e**).

Eleven species were considered to be vent specific (four described and seven awaiting description), all of which being recorded exclusively from LCVF to date. Formal species descriptions of the new species will be published in separate papers. Five species from the LCVF were previously known from cold seeps. Four of these are annelids, including the two widely distributed species *Sclerolinum contortum* and *Nicomache lokii*, which are known from cold seeps in the Arctic, Gulf of Mexico and Barbados, and sedimented vents in the Southern Ocean^34,35^ (Fig. 3a and e). The species *Oligobrachia* sp. “Vestnesa”, an undescribed species only previously recorded from the Vestnesa cold seep^36^, was collected in 2022 from the Oasis area of LCVF (Fig. 3b). The tubes of *Oligobrachia* sp. “Vestnesa” were found intertwined with *Sclerolinum contortum*, but the former species could be distinguished by straighter tubes. The identity of *Oligobrachia* sp. “Vestnesa” from LCVF is supported by DNA barcodes of the COI gene (Supplementary Table S1). At the collection site, *Sclerolinum contortum* outnumbered *Oligobrachia* sp. “Vestnesa” by at least an order of magnitude, but a proper quantification of their relative densities was not possible due to difficulties with confidently telling the worms apart without extracting them from the tube. The final seep-associated annelid recorded from the LCVF is the cirratulid *Raricirrus arcticus*, which was originally described from a locality near the Gakkel Ridge in the Laptev Sea believed to be a cold seep^37^. The fifth species previously recorded from cold seeps is the gastropod *Rissoa griegi*, which was originally described from a wood fall (discussed below). *Rissoa griegi* is also known from the shallower hydrothermal vents of the Jan Mayen Vent Field (as *Rissoa* cf. *griegi*)^16^ and it has been claimed that the same species is found in the Nyegga cold seeps^1,16^, but this record has not been documented with morphological or molecular data in published literature.

Four species from the LCFV were previously known from wood falls, the annelid *Sclerolinum contortum* and the gastropods *Rissoa griegi, Pseudosetia semipellucida* and *Skenea profunda* (Fig. 3g and i). The three gastropods were all described from a piece of sunken wood colonised by wood-boring bivalves at 2,437 m depth west of Svalbard^38^. *Rissoa griegi* has been considered a junior subjective synonym of the shallow water species *Pusillina tumidula*^39^, but this synonymisation has subsequently been retracted^40^. *Skenea profunda* has recently been recorded from a cold seep in the central Arctic Ocean^41^, and the present records from the LCVF demonstrate that *S. profunda* is also able to inhabit hydrothermal vents.

The Barite Field has the highest species richness of the different areas in the vent field with 33 taxa recorded, and most of these are found in the worm forests. Oasis, the other diffuse-venting area, has a lower taxon richness (19 taxa), but this area was only recently discovered in 2018 and has only been sampled during three cruises (2019, 2020 and 2022). Seventeen taxa were recorded from the chimneys and surrounding areas on the mounds, and these are largely a subset of the taxa found in the diffuse venting areas. The only taxa that were recorded exclusively from the mounds are two actiniarians in the family Kadosactinidae, the amphipods *Cleippides quadricuspis* and Calliopidae indet. and an unidentified species of Solenogastres (Table 1). The amphipods are part of the background fauna, and from video observation we can confirm that at least *C. quadricuspis* is commonly found in the Barite Field as well (see Fig. 2d). The only species that were sampled from the black smoker chimneys, near the high temperature venting, are the gastropods *Rissoa griegi* and *Skenea profunda*, and the amphipod *Exitomelita sigynae*.

### Stable isotope analysis

We aquired stable isotope values for 22 animal taxa from LCVF, and a sample of a bacterial mat from a black smoker chimney on the western mound. Some taxa could be identified only to higher taxonomic levels prior to analysis, such as the Actiniaria and Nematoda. In some cases, there was additional diversity within a taxon that was not discovered until after the sampling for isotopes was completed. This was the case for Calliopiidae indet., for which we do not know if the sampled individuals belong to the new species of Calliopiidae or the second species. It is also unknown if the samples of *Ophryotrocha* spp. nov. contain individuals from one of the two new species, or a mix of both. Between 1 and 8 samples were analysed for each taxon, of which some samples were composed of multiple individuals pooled due to small body size (see Table 2). The resulting stable isotope values from the gastropod *Mohnia mohni* showed two very divergent clusters, which were divided in the table and highlighted in the figure for clarity; group A with heavier values of both δ^15^N and δ^13^C, and group B with lighter values (Table 2). The full table of isotope values can be found in Supplementary Table S2.

**Table 2:** Overview table of stable isotope measurements.

Taxon	Average δ^15^N	SD N	Average δ^13^C	SD C	N	Ind/sample
Bacterial mat	− **13,70**	**1,26**	− **19,67**	**3,22**	**3**	**–**
*Caulleriella* sp. nov	3,13	0,58	− 27,54	0,23	3	1
Lumbrineridae gen. et*.* sp. nov	2,33	0,10	− 24,84	0,79	3	1
*Nicomache lokii*	2,64	1,59	− 26,59	0,57	2	1–2
*Notomastus* sp. nov	4,47	0,97	− 27,27	1,02	3	1
*Ophryotrocha* spp. nov	− 3,34	1,00	− 23,91	2,45	3	1
*Orbiniella* sp. nov	2,94	NA	− 26,55	NA	1	3
*Paramytha schanderi*	− 0,70	0,61	− 28,49	0,53	3	1
*Phisidia* sp. nov	− 0,39	NA	− 28,30	NA	1	3
*Raricirrus arcticus*	0,79	0,58	− 27,08	0,63	3	1
*Sclerolinum contortum*	− 7,53	0,96	− 25,89	0,38	5	1
Calliopiidae indet	− 0,55	0,56	− 24,18	0,62	2	1
*Exitomelita sigynae*	− 5,93	0,97	− 23,97	2,17	3	1
*Laothoes* sp.	0,03	NA	− 22,45	NA	1	1
*Monoculodes bousfieldi*	0,06	0,56	− 23,48	1,34	5	1
*Paroediceros lynceus*	2,91	2,99	− 15,32	2,78	3	1
*Seba armata*	− 0,48	NA	− 23,03	NA	1	1
*Mohnia mohni* Cluster A	13,14	1,84	− 19,40	0,77	8	Part-1
*Mohnia mohni* Cluster B	− 3,12	1,75	− 36,90	2,62	4	Part-1
*Rissoa griegi*	− 3,34	0,95	− 25,80	0,56	6	3–5
*Bythocaris leucopis*	3,64	0,08	− 21,32	0,03	3	Part
Actinaria	1,08	0,46	− 31,86	0,54	6	Part
Zoarcidae indet	2,89	1,24	− 21,80	1,58	2	Part
Nematoda	− 1,07	0,05	− 21,45	0,09	3	3

Average stable isotope values for each taxon are shown (as ‰), standard deviation (SD), number of samples analysed (N) and individuals per sample.

### Context for stable isotope interpretation

δ^13^C values can be indicative of the carbon fixation pathway of the primary producers in the foodweb, but the isotopic signatures of different pathways are variable depending on several factors (e.g. local isotopic baselines or temperature)^42,43^. The stable isotope (SI) signatures of basal sources of organic matter (e.g. POM, microbial communities) in the foodweb have not been characterised at LCVF, except for the single bacterial mat included in this analysis. In addition, the low δ^13^C values of CO_2_ and CH_4_ in the vent fluids at LCVF (CO_2_: − 13.4 to − 11.3 ± 0.1‰, CH_4_: − 29.1 ± 0.3‰)^7^ are quite different to what is commonly found in other vent fields^44^. This means that we cannot use standard δ^13^C values for different microbial fixation pathways from the literature. To illustrate typical SI signatures of photosynthesis-derived particulate organic matter (POM) in the Arctic, we used SI values from the literature of sediment POM (sPOM) from similar depth as the LCVF and pelagic POM (pPOM) from surface waters (Supplementary Table S3). sPOM from areas without chemosynthetic influence in the Arctic region between 2000 and 2800 m depth showed δ^13^C values from − 21.35 to − 23.5‰ and δ^15^N from 5 to 8.62‰^45–47^. pPOM from the Barents Sea and Norwegian Sea showed δ^13^C values from − 22 to − 27.3‰ and δ^15^N from 4 to 5.4‰^48–50^.

**Figure 4:**
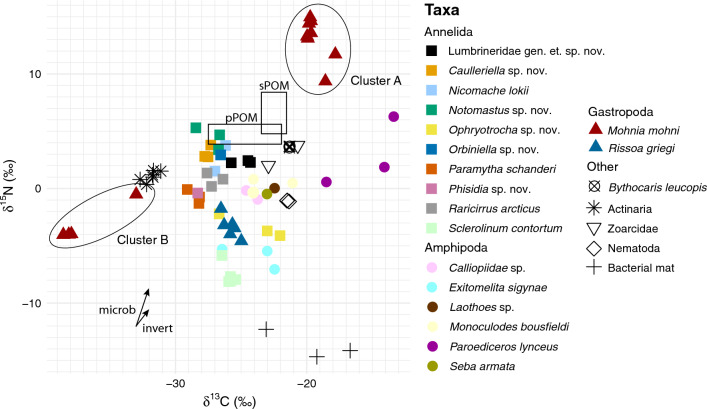
Stable isotope values of each analysed sample. δ^13^C on the X-axis and δ^15^N on the Y-axis, both in ‰. Each taxon is coloured with a unique colour, and higher taxon groups are indicated by the shape of the point. The two distinct clusters of *Mohnia mohni* (Cluster A and B) are enclosed in ellipses. Isotopic signatures of photosynthesis-derived POM from sediment (sPOM) and pelagic samples (pPOM) from the literature are indicated by open squares. The assumed isotopic enrichment per trophic level of microbial feeders (microb) and invertebrate feeders (invert) is indicated with arrows.

δ^15^N can be used to indicate the trophic level of organisms, because marine food webs typically show an enrichment of δ^15^N per trophic level. While a standard rate of + 3.5‰ change in δ^15^N per trophic level has frequently been used, this value is not suitable for organisms with an invertebrate diet^51^. Here we assume a trophic shift of + 1.4‰ for invertebrate diets and + 3.3‰ for microbial diets (indicated in Fig. 4)^52^. A smaller enrichment per tropic level is also seen in δ^13^C, typically 1‰ (or as low as 0.3‰ for insects/crustaceans with chitinous exoskeletons)^51,52^.

### Stable isotope results

Only one bacterial mat sample was available for analysis of isotopes, which was collected from a black smoker chimney on the western mound. The bacterial mat sampled shows very variable δ^13^C signature between subsamples, which could be due to different proportions of bacterial groups in the subsamples. The δ^13^C signature of the bacterial mat was somewhat more enriched than photosynthesis-derived matter (− 17 to − 23‰), and it has the most depleted δ^15^N values in the dataset (average − 13,70‰).

The median δ^13^C of all the fauna analysed was − 25.6‰ (see Table 2 for averages for each taxon), which falls within the range of pPOM values reported in the literature, but is more depleted than sPOM. However, nearly all the faunal samples had depleted δ^15^N values compared to sPOM and pPOM, indicating that photosynthesis-derived POM is not a dominating food-source for this community. Most of the amphipods clustered together (except *Exitomelita sigynae* and *Paroedicerus lynceus*), and displayed slightly heavier δ^13^C values than the main polychaete cluster (between − 20 and − 25‰). The highly variable isotope values of both nitrogen and carbon from the gastropod *Mohnia mohni* was unexpected since the sampled individuals were morphologically identified as belonging to the same species. However, the two clusters were collected from different parts of the vent field, which might explain the observed differences (see Discussion). Cluster A of *Mohnia mohni* had δ^13^C close to − 20‰, while Cluster B had the most depleted δ^13^C values of the dataset (average δ^13^C − 36.9‰).

The lightest nitrogen values in the faunal samples from LCVF were found in the two known symbiotrophic organisms from LCVF, *Sclerolinum contortum* and *Exitomelita sigynae*^20^ (Fig. 4). The gastropod *Rissoa griegi* and the polychaetes *Ophryotrocha* spp. nov. had slightly heavier nitrogen values than the symbiotrophic species, but still lighter than the main cluster of samples. Most of the remaining taxa had nitrogen values between approximately 0–5, which could indicate feeding by bacterivory, deposit feeding, scavenging or predation on the organisms with lighter nitrogen values.

## Discussion

In this paper, we present a comprehensive species inventory of the Loki’s Castle vent field (LCVF) on the Arctic Mid-Ocean Ridge. The fauna of LCVF has a high degree of novelty. Five species have previously been described from this locality^18–21^, and here we record another ten species that are considered new to science and are awaiting formal description. Most of the new species from LCVF are hydrothermal vent specialists, and the vent field also hosts some species known from other chemosynthesis-based ecosystems in the region. This demonstrates that the LCVF is home to a clearly specialised fauna, in contrast to what is known from the shallower Jan Mayen vent fields further south on the Mohns Ridge^16^. Some of the new and undescribed species discovered in LCVF have also been found in samples from the non-vent deep sea, such as *Caulleriella* sp. nov., *Orbiniella* sp. nov. and Lumbrineridae gen. et. sp. nov. (see Supplementary Notes for more detail). This highlights that there is undescribed diversity in the deep Nordic Seas also outside of chemosynthesis-based ecosystems. Another interesting observation is that some taxa were only collected in very low numbers, indicating that they are either genuinely very rare or exhibit a very patchy distribution. One extreme example is the *Anobothrus* sp. nov. (Ampharetidae), which was represented only by a single specimen collected in 2015. This was, however, a large-sized well-preserved individual that could be used for both molecular and morphological analysis and was identified as a new species. Similarly, the finding of *Oligobrachia* sp. “Vestnesa” at LCVF in 2022 highlights the benefit of repeated sampling to properly characterise such patchy environments as hydrothermal vents. In the case of taxa that are usually most abundant in the meiofaunal size fraction, such as the nematodes, the rarity of certain taxa is probably due to the sampling methods not capturing the meiofauna.

The fauna of LCVF is highly distinct compared to that of hydrothermal vents in other oceanic regions. The only species shared with hydrothermal vents on the Mid-Atlantic Ridge (MAR) is the hydrozoan *Hydrallmania falcata.* This species is common in shallow waters in the northern Atlantic Ocean, and has been recorded from the Lucky Strike hydrothermal vent^53,54^. The specimen from LCVF is morphologically indistinguishable from colonies collected in shallower waters^54^, but an integrated analysis with molecular data is required to evaluate the status of the deep-water vent-associated population. The two widely distributed species *Sclerolinum contortum* and *Nicomache lokii* are shared between LCVF, cold seeps in the Gulf of Mexico and Barbados, and vents in the Southern Ocean^34,35^. However, there is some genetic differentiation between these very distant populations, and it is unclear to which degree there is present-day geneflow between them^34^. Preliminary data on the faunal community of the Aurora vent field on the Gakkel Ridge in the Arctic Ocean indicates some similarities with the fauna of LCVF (rissoid and skeneid gastropods, and melitid amphipods)^23^, but also revealed a recently described species of cocculinid gastropod^55^, which is not known from LCVF. However, species level identification of the fauna from Aurora is needed to assess the similarity between the faunal communities of these two Arctic vent sites. The faunal inventory presented here makes it possible to test the hypothesis that the AMOR constitutes a distinct biogeographical province by including the Arctic in a biogeographic analysis of the global vent fauna. This work is in progress and will be presented in a separate paper.

At a higher taxonomic level, there are several taxa at the LCVF that are common in hydrothermal vents globally. The maldanid genus *Nicomache* is well known from hydrothermal vents in the Pacific^56^, *Ophryotrocha* species are abundant in hydrothermal vents and cold seeps in all oceans^57^, and Ampharetidae have several clades adapted to hydrothermal vents, cold seeps and organic falls^58^. Several species of amphipods in the family Oedicerotide are known from hydrothermal vents on the MAR, including *Monoculodes anophtalma*^59^*.* However, some of the dominant fauna of the LCVF belong to taxa not typically recorded from hydrothermal vents in other oceans, such as the family Rissoidae and the genus *Skenea*^60^ in the Gastropoda and the amphipod family Melitidae^20^. This could indicate a regional adaptation pathway, possibly from fauna associated with other CBEs such as cold seeps and organic falls. Both *Rissoa griegi* and *Skenea profunda* were originally described from sunken wood^38^, and the amphipod genus *Exitomelita* has only two described species—*E. sigynae* from LCVF and the closely related *E. lignicola* collected from a wood fall nearby^61^. The close connection between hydrothermal vent and wood fall fauna in the Arctic was also in the description of the vent limpet *Cocculina aurora*, which belongs to a genus mainly associated with sunken wood^55^.

Several of the common taxa at the LCVF belong to clades that are in urgent need of taxonomic revision, which hinders our understanding of biogeographic patterns and evolutionary history. One example is the gastropod *Rissoa griegi*, which is extremely abundant at LCVF, but has an uncertain generic assignment. A recent molecular phylogeny revealed that several genera in the Rissoidae are not monophyletic^62^, and preliminary sequence analyses using the BOLD database shows that *R. griegi* and *Pseudosetia semipellucida* do not cluster together with *Pseudosetia turgida* (type species of *Pseudosetia*) or other *Rissoa* spp. Phylogenetic analyses including both mitochondrial and nuclear markers are needed to resolve the generic placement of these two species. The gastropod family Skeneidae has also been under recent taxonomic revision based on molecular data^63^, but the nominal genus *Skenea* has not been included in any molecular phylogenetic analyses to date, and prior to the present paper there were no sequences of *Skenea* available in NCBI Genbank or BOLD. Additionaly, eelpouts (Zoarcidae) represent another case of a family with unresolved taxonomic issues, as a recent phylogeny showed that many of the genera are not monophyletic^64^. The most abundant eelpout at LCVF has proved challenging to identify to the genus level both using both morphological keys and DNA barcoding. Thus, for the purpose of this paper this eelpout was left at Zoarcidae indet. Some of the characteristics necessary to distinguish species and genera of eelpouts (e.g. scale coverage or pectoral fin characteristics) are often not available due to damage inflicted during the sampling process or the small size of the sampled individuals. However, there are ongoing efforts to resolve the taxonomic position of this species using an integrated morphological and molecular approach.

The worm forests of the diffuse venting areas at LCVF (Barite Field and Oasis) are a unique biogenic habitat that appears to promote biodiversity in the vent field, as demonstrated by the higher taxon richness recorded in these areas. In contrast to foundation species at vents and seeps in other oceans, which typically have a large body-size such as the “giant tubeworms” found at Pacific hydrothermal vents^65^, the Arctic tubeworms *Sclerolinum contortum* and *Oligobrachia* sp. “Vestnesa” form long and thin tubes (a few mm in diameter) that anchor in sediments (see Fig. 2h). The maldanid worm *Nicomache lokii* also contributes to the structure of the worm forests by forming a dense mat of tubes at the base of the *S. contortum* bushes^19^. While dense patches of *Sclerolinum contortum* and *Oligobrachia* spp. are also common in cold seeps in the Arctic, associated communities are mainly composed of background fauna^66^. However, most of the cold seeps studied in the Arctic are found in shallower waters, which are also known from other ocean regions to have a less specialised fauna^67^. The high prevalence of specialised fauna at LCVF compared to the shallower JMVF suggests that it is important to study deeper cold seeps to test whether these might have a higher degree of specialised fauna.

The isotopic signatures of the vent community of LCVF show a clear influence of chemosynthetic primary production on the foodweb, supported mainly by the depleted δ^15^N values of the whole community. Low, even negative, values of δ^15^ N are often found in vent fauna, especially symbiotrophic organisms^68,69^. Depleted δ^15^N values indicate that there is local fixation of inorganic nitrogen^70^, or possibly bacterial uptake of ammonium, which is abundant in the vent fluids at LCVF^9,71^. Isotope signatures of fauna from the shallower JMVF showed mainly reliance on photosynthetically derived organic matter, with a few exceptions^72^. This is consistent with the hypothesis that the fauna of JMVF is less specialised than at LCVF because of a higher availability of photosynthetically derived organic matter in the former vent sites, reducing the evolutionary pressure to adapt to the harsh conditions in the areas where chemosynthetically derived organic matter is most abundant^16^. However, it can not be excluded that some of the differences in isotopic signatures of fauna from LCVF and JMVF are attributable to different isotopic baselines of the two vent fields, as has been shown for other vent fields^68^.

Deciphering the contribution of various sources of carbon to the foodweb at LCVF is challenging due to incomplete characterisation of SI signatures of the microbial primary producers at its base. In addition, the isotopic signatures of the dissolved carbon sources in the vent fluids are impacted by sediment-fluid interactions, leading to low δ^13^C values of both CO_2_ and CH_4_^7^. The local methane δ^13^C values at LCVF lie close to those of photosynthetically derived organic matter, further confounding the patterns. Our understanding of resource partitioning at the LCVF could be improved in future studies by characterising the isotopic signatures of the local basal resources, and by including sulphur isotopes (δ^34^S)^73^.

The SI signatures of the sampled species generally correspond well with the available data on their trophic ecology. The lowest δ^15^N values are found in the symbiotrophic species *Sclerolinum contortum* and *Exitomelita sigynae*, which is a common pattern in hydrothermal vent foodwebs^44,52,68^. The SI signature of *E. sigynae* in this study is similar to what was reported in the original description of the species (δ^13^C − 23.0‰ in the description paper vs. − 24.0‰ in this study, δ^15^N − 5.9‰ in both studies), where it was hypothesised to have a combined feeding ecology with bacterial grazing as well as ectosymbionts on the gills^20^. *Rissoa griegi* and *Ophryotrocha* sp. nov. have higher δ^15^N values than the symbiotrophic species, but lower than the remaining samples. This could indicate that *R. griegi* and *Ophryotrocha* sp. nov. are feeding lower in the foodweb than the other taxa analyzed, for example because of a more selective bacterial feeding. Isotope analyses of *R. griegi* (as *Pseudosetia griegi*) from the Jan Mayen vent fields showed high similarities between the gastropods and sampled microbial communities, supporting that they are bacterivores^72^. *Ophryotrocha* sp. are known as bacterivores, and co-occuring species appear to be able to feed on distinct microbial resources, thus reducing competition between species^74^. It should be noted, however, that lab experiments on the incorporation of organic matter from various sources by *Ophryotrocha labronica* have revealed that the isotope tissue-diet shifts for both δ^13^C and δ^15^N were very variable and dependent on the food source, and that the δ^15^N shift could even sometimes be negative^75^.

Most of the remaining species sampled have δ^15^N values between 0 and 5‰, and may be feeding on microbial communities, detritus or a combination of both. The amphipods (except *E. sigynae*) have somewhat heavier δ^13^C than the main cluster of polychaetes, which may indicate that they are consuming a comparatively higher proportion of phytodetritus. The maldanid polychaete *Nicomache lokii* is believed to be a grazer consuming a combination of microbial communities and phytodetritus, and isotope data from the original description paper were similar to the data presented here (δ^13^C = − 22.5 and δ^15^N = 3.8 vs. δ^13^C = − 26.6 and δ^15^N = 2.6). Interestingly, *N. lokii* from cold seeps off Barbados showed widely variable SI values indicating a generalist deposit-feeding strategy, but chemosynthesis-derived detritus appears to be an important component of the diet^76^.

Larger predator species in the LCVF vent community are represented by the eelpout fish (Zoarcidae indet.), the actiniarians and the buccinid gastropod *Mohnia mohni*. Based on the isotope values and direct observations of gut content, the eelpout is inferred to feed on amphipods from the main cluster (excluding *E. sigynae* and *P. lynceus*). The only taxon with isotope values that matches what one would expect from the prey of the actiniarians is *Mohnia mohni* (cluster B), but this is unlikely given the motility and large size of *M. mohni*. It seems more likely that the prey of the actiniarians is an unsampled taxon with a similar isotopic signature as *M. mohni.* The only polychaete family at LCVF known to dispaly a predatory lifestyle is the Lumbrineridae, represented by Lumbrineridae gen. et sp. nov. This species has slightly more enriched carbon values than the other polychaetes, which could reflect its prey. Recent studies with stable isotopes have generally confirmed carnivory in lumbrinerids but some species seem to utilise detritus and rapid uptake of δ^13^C-labelled diatom detritus has been demonstrated in field experiments from continental slope and deep fjord environments^77^. The δ^13^C values could be due to a larger proportion of photosynthetically derived organic matter in the diet of Lumbrineridae gen. et sp. nov compared to the other polychaetes. *Bythocaris* shrimp are known as scavengers^78^, and field observations from baited traps and colonization experiments with wood and bone substrates near LCVF supports this. *Bythocaris leucopis* is probably an opportunistic feeder, and the relatively enriched δ^15^N values support that it consumes animal tissue, at least as part of its diet.

The samples of *M. mohni* from LCVF cluster in two highly divergent groups, displaying the most enriched δ^15^N values (Cluster A) and the most depleted δ^13^C values (Cluster B) of the whole dataset. The sampled individuals were morphologically identified as belonging to the same species, but they were sampled in different areas of the vent field. Specimens in Cluster B were sampled on the western mound, and Cluster A probably in the Barite Field, although the exact position remains uncertain. Buccinids are usually considered predators or scavengers, and the enriched δ^15^N level of *M. mohni* Cluster A could support such a predatory lifestyle (Fig. 2), but there are no sampled taxa that match the isotope signature expected of their prey. *Mohnia mohni* Cluster B, however, have more depleted δ^15^N values, suggesting that it feeds at a lower trophic level, or on prey with very depleted δ^15^N. Thus, it is possible that *M. mohni* is a generalist, and is feeding on different food-sources with distinct isotopic signatures in the various areas of the vent field, depending on food availability. An alternative hypothesis is that there is more than one ecotype of *M. mohni* with different feeding preferences^79^, which could indicate cryptic or early-stage speciation. These hypotheses should be tested with combined analysis of stable isotopes, gut content and DNA barcoding of *M. mohni* from various areas within the vent field.

Comparison of stable isotope signatures of some species from LCVF with conspecifics from other locations reveals a high intraspecific variability. In the JMVF, isotope signatures of *Rissoa griegi* (as *Pseudosetia griegi*), indicates that *R. griegi* feeds on different microbial groups in high temperature and low temperature microhabitats^72^. Average δ^13^C and δ^15^N of *R. griegi* from a high temperature venting area in the JMVF were − 34.0 and 3.1‰, respectively, while for a low temperature area the δ^13^C was less depleted (− 20.8‰)^72^. The samples of *R. griegi* analysed here were all collected from the Barite Field diffuse venting area, and thus we cannot confirm if the same intra-field variation for this species is found at LCVF. However, the isotopic signature of *R. griegi* from the Barite Field differs from both the high- and low-temperature habitats at JMVF (average δ^13^C − 25,80 and δ^15^N − 3,34‰). The variation in SI signatures could indicate a high degree of trophic flexibility in *R. griegi*, but may also reflect different isotopic baselines in the two vent fields. *Sclerolinum contortum* also shows a high variability in SI signatures between locations. Populations from the Arctic cold seep Haakon Mosby Mud Volcano (HMMV) have more depleted δ^13^C values than the samples from LCVF (from − 34.9 to − 48.3 at HMMV vs. − 25.4 to − 26.5 at LCVF)^80^. At the Hook Ridge hydrothermal vents in the Southern Ocean, on the other hand, *S. contortum* has more enriched δ^13^C values than both the former sites (average − 20.5‰)^81^. *Sclerolinum contortum* is a symbiotrophic species, relying on nutrients from sulphide oxidising bacteria hosted in a specialised organ^82^. The symbiont of *S. contortum* has so far been characterised only in specimens from HMMV, but assuming that it is the same across locations with the same metabolic pathway and isotopic fractination pattern, the discrepancy in δ^13^C values could still be explained by differences in δ^13^C of the local carbon sources. For example, methane at HMMV has δ^13^C of − 60‰^81^, while at LCVF it is much less depleted with values from − 27 to − 29‰^7^.

### Conclusions and implications for management

The diverse and specialised vent community presented here from LCVF illustrates the vulnerability of the vent community to disturbances from industrial activities. The dependence of the vent-endemic fauna on a habitat that exists only in a very small area implies that destructive disturbances in these areas could lead to regional or even global species extinctions^28^. For the taxa at LCVF that are known from other Arctic CBEs, the number of geographic records of each species is very low. There is an urgent need for better understanding of the distribution, habitat selectivity and genetic connectivity of fauna from CBEs in the Arctic region. Because CBEs are patchily distributed, the specialised species are dependent on dispersal between sites with suitable environmental conditions to maintain metapopulation connectivity, and disruption of connectivity could lead to impacts beyond a single affected site by isolating adjacent populations^27^.

Efforts are ongoing to describe the new species found at LCVF, and this is an important task from a management perspective. Six species from the LCVF are listed as vulnerable in the Norwegian Red List for Species^83^, and these species are also under consideration for the newly started assesment of vent-endemic fauna for the IUCN. However, unnamed species are not included in red list evaluations, highlighting the importance of dedicating sufficient resources to deep-sea taxonomy. In addition, the meiofaunal size fraction (< 0.5 mm) of the fauna of LCVF has not been explored yet and is generally poorly known from CBEs in the region^84^. Internationally, active hydrothermal vents are considered vulnerable marine ecosystems (VMEs), but in the Norwegian Red List for Nature Types, hydrothermal vents are ranked as habitats of least concern^85^. Considering the novel and specialised fauna documented in this paper, hydrothermal vents on the AMOR should be regarded as VMEs and measures must be implemented to protect them from direct and indirect impacts of potential deep-sea mining activities.

## Methods

### Sample collection and documentation

Samples from LCVF were collected during cruises on the RV GO Sars in 2008, 2009, 2010, 2015, 2017, 2018, 2019, 2020 and 2022 organised by the Centre for Geobiology (2008–2017), KG Jebsen Centre for Deep Sea Research (2017–2020) and Centre for Deep Sea Research (2022). In addition, some samples from inactive areas in the vicinity of LCVF (collected in 2007–2009) was included to document the presence of certain species outside the vent field. A full list of sampling stations can be found in supplementary Table S4. The samples were collected with the ROVs Bathysaurus (2008–2014; ARGUS Remote Systems) and Ægir6000 (2015–2022; Kystdesign), using the ROV arm, suction sampler and various scoops and coring devices (e.g. pushcorer, bladecorer). On board the ship, samples were sieved carefully through a series of sieves with mesh size > 0.5 mm and fixed in 96% molecular grade ethanol for barcoding and selected specimens in 10% buffered formalin solution for morphological study. Images of live specimens were taken on board using a Canon EOS 5D MarkII or a LabCam Microscope Camera (iDu Optics) equipped with the iPhone6S (Apple) on a Leica MZ 9.5 stereomicroscope (Leica Microsystems). Ethanol fixed specimens were photographed in the lab using a Leica M205c stereomicroscope with Leica DMC 5400 camera and Leica Application Suite (LAS 4.13), stacked with Zerene stacker 1.04. In situ images are screen captures from high-definition video collected by the ROV Ægir6000. The overview map (Fig. 1a) was generated in R^10^ using the ggOceanMaps package^11^, which utilised bathymetry from NOAA^12^ and land polygons from Natural Earth Data^13^. The bathymentric map of the Loki’s Castle vent field (Fig. 1b) was generated using QGIS^14^ and GMT^15^.

### Morphological identification

Benthic cnidarians (Hydrozoa, Actiniaria) were identified using a combination of external and internal morphological characters as well as detailed analysis of the cnidome. Polychaete worms were identified using stereo and compound microscopes for external morphological and chaetal characters. A minimum of 20 Nematoda specimens preserved in 96% ethanol were picked out randomly from each sample and mounted on permanent glycerin slides for morphological analyses, while an additional set was mounted on temporary slides with distilled water for DNA barcoding^86^. Nematodes were then identified under the microscope, using available literature^87–89^. All nematode specimens belonged to the macrofauna size fraction only (> 0.5 mm). Gastropods were identified based on external morphology of the shell and soft parts, polychaetes were identified based on external morphology, and both were compared to literature and reference material from the collections of the University Museum of Bergen. Hyperbenthic copepods (Calanoida, Harpacticoida, Cyclopoida) were identified based on morphological characters, according to standard keys and literature^90–93^. A non-destructive DNA-extraction was performed to retain the copepod exoskeletons, which were mounted on permanent slides (Hydro-Matrix medium). Benthic Amphipoda (Crustacea, Peracarida) were identified morphologically using available taxonomic literature. Permanent slides (Faure’s medium) were made for new species.

Some taxonomic groups were identified only to higher taxonomic levels due to the condition of the samples or lack of taxonomic expertise. In the final dataset of species occurrences, taxonomic units that were identified to a taxonomic level higher than species were only included if they were morphologically distinct from other specimens of the same taxa. This was done to enable inclusion of specimens identified to higher taxa without overestimating taxon richness. Voucher material documenting the identified taxa has been deposited in the collections of the University Museum of Bergen and a dataset of georeferenced species records has been uploaded to GBIF (see Data availability).

### DNA barcoding

Extraction of total DNA was performed using the QuickExtract™ DNA Extraction Solution Kit (Epicentre Biotechnologies) or the QIAGEN DNeasy Blood and Tissue Kit following the manufacturers protocol. The genetic marker used depended on the taxonomic group, and the protocols that were available with good success rates. PCR primers and protocols used for each taxon group are available in Supplementary Table S5. Quality and quantity of amplicons were assessed by gel electrophoresis imaging using a FastRuler DNA Ladder (Life Technologies) and GeneSnap and GeneTools (SynGene) for image capture and band quantification. Successful PCRs were purified using Exonuclease 1 (EXO, 10 U mL^−1^) and Shrimp 90 Alkaline Phosphatase (SAP, 10 U mL^−1^, USB Europe, Germany) in 10 μL reactions (0.1 mL EXO, 1 μL SAP, 0.9 μL ddH 2O, and 8 μL PCR product). Samples were incubated at 37 °C for 15 min and inactivated at 80 °C for 15 min. The purified PCR products were sequenced using BigDye v3.1 (Life Technologies) and sequenced on an Automatic Sequencer 3730XL at the sequencing facility of the Department of Biological Sciences, University of Bergen. Alternatively, PCR products were sent for purification and sequencing by Macrogen Europe (Amsterdam, The Netherlands).

Forward and reverse sequences were assembled, and quality controlled using the software Geneious (Biomatters Ltd.) and compared with available reference sequences in the BOLD database and NCBI Genbank. GenBank voucher numbers for each taxon are available in Supplementary Table S1.

### Stable isotope analyses

Stable isotope analyses (δ^13^C and δ^15^N) were conducted at the University of Bergen, using material preserved on 96% ethanol. For large-size animals, such as fish, tissue was subsampled from clean muscle-tissue (Supplemenary Table S2). For small animals, whole animals were used, and for very small animals 3–5 individuals were pooled in one sample to obtain enough tissue for analysis. Approximately 1 mg of dried tissue was used for analysis. Where possible, three or more replicates were included for each taxon.

The samples were prepared by removing gastropods from their shell, and polychaetes from their tubes, if possible, and then placed in an oven to dry at 80 °C for 24 h in glass vials and ground using a glass pestle. To remove lipids from the samples, 200 μg of dichloromethane was added, and after two hours, the liquid was pipetted away, and the sample was again dried at 80 °C for 24 h. Inorganic carbon, particularly calcium carbonate from gastropods, was then removed by adding 200μg of 0.1M HCl. Depending on the amount of inorganic carbon present in each sample the treatment time with 0.1M HCl varied from a minimum of 5 min, or until the reaction was finished. After the treatment the samples were rinsed repeatedly with ddH2O until pH reached 6–7. The HCl treatment was followed by drying the samples overnight at 80 °C, and then weighing them together with the pre-weighed tin capsules. Stable isotopes were measured using Delta V Plus isotope ratio mass spectrometer connected to a Flash EA 1112 elemental analyser (Thermo Scientific). Isotope ratios are expressed in delta notation as‰ difference in ^13^C/^12^C and ^15^N/^14^N isotope ratios compared to Pee Dee Belemnite (PDB) and air N2, respectively. Samples were calibrated to internationally acknowledged C and N isotope ratio standards.

Ethanol preservation of tissue can have an effect on δ^13^C and δ^15^N signatures, but this effect is not consistent between studies and generally of low magnitude compared to the natural variability of marine food sources^44,94^. Therefore, we did not use any correction for preservation on the isotope data. Samples for stable isotope analyses were collected between 2008 and 2010, and analyses were performed in 2016–2017, and thus the storage time in ethanol was similar for all samples. The isotope data was plotted in R using the packages ggplot2 and tidyverse, and final graphical adjustments were done in Adobe Illustrator. The R-script for the isotope plot is available in Supplementary Methods.

### Supplementary Information

Supplementary Information 1.

Supplementary Tables.

## Data Availability

Further taxonomic and ecological notes for each taxon is available in Supplementary Notes. Georeferenced species occurrence records will be shared via GBIF as part of the dataset “Invertebrate collections, University Museum of Bergen” (https://doi.org/10.15468/f2y3bf) and “Ichtyological collections, University Museum of Bergen”, and all the records that form the basis for the taxon list in this paper is available in darwincore format through Figshare (https://doi.org/10.6084/m9.figshare.24454108). DNA sequences supporting the identification of selected taxa will be released in BOLD and Genbank, and accession numbers are available in Supplementary Table S1. Raw data and R-script for the isotope analysis is available in Supplementary Table S2 and Supplementary Methods.

## References

[1] Pedersen RB (2010). Discovery of a black smoker vent field and vent fauna at the Arctic Mid-Ocean Ridge. Nature Commun..

[2] Dick GJ (2019). The microbiomes of deep-sea hydrothermal vents: distributed globally, shaped locally. Nat. Rev. Microbiol..

[3] McMullin ER, Bergquist DC, Fisher CR (2007). Metazoans in extreme environments: Adaptations of hydrothermal vent and hydrocarbon seep fauna. Gravit. Space Res..

[4] Sogin EM, Leisch N, Dubilier N (2020). Chemosynthetic symbioses. Curr. Biol..

[5] Kiel S (2016). A biogeographic network reveals evolutionary links between deep-sea hydrothermal vent and methane seep faunas. Proc. Roy. Soc. B Biol. Sci..

[6] Pedersen, R. B., Thorseth, I. H., Nygård, T. E., Lilley, M. D. & Kelley, D. S. Hydrothermal activity at the arctic mid-ocean ridges. In *Diversity of Hydrothermal Systems on Slow Spreading Ocean Ridges* 67–89 (American Geophysical Union (AGU), 2010). 10.1029/2008GM000783

[7] Baumberger T (2016). Fluid composition of the sediment-influenced Loki’s Castle vent field at the ultra-slow spreading Arctic Mid-Ocean Ridge. Geochim. Cosmochim. Acta.

[8] Eickmann B (2014). Barite in hydrothermal environments as a recorder of subseafloor processes: A multiple-isotope study from the Loki’s Castle vent field. Geobiology.

[9] Steen IH (2016). Novel Barite Chimneys at the Loki’s Castle Vent field shed light on key factors shaping microbial communities and functions in hydrothermal systems. Front. Microbiol..

[10] R Core Team. R: A language and environment for statistical computing. *R Foundation for Statistical Computing, Vienna, Austria.* https://www.R-project.org/ (2022).

[11] Vihtakari, M. ggOceanMaps: Plot Data on Oceanographic Maps using ‘ggplot2’. R package version 2.1.1. https://mikkovihtakari.github.io/ggOceanMaps/ (2023).

[12] NOAA National Centers for Environmental Information. ETOPO 2022 15 Arc-Second Global Relief Model. 10.25921/fd45-gt74 (2022).

[13] Natural Earth. 1:10m Physical Vectors. https://www.naturalearthdata.com/downloads/10m-physical-vectors/ (2023).

[14] QGIS. QGIS Geographic Information System. Open Source Geospatial Foundation Project. Version 3.32. https://www.qgis.org/ (2023).

[15] Wessel P (2019). The generic mapping tools version 6. Geochem. Geophys. Geosyst..

[16] Schander C (2010). The fauna of hydrothermal vents on the Mohn Ridge (North Atlantic). Mar. Biol. Res..

[17] Tarasov VG, Gebruk AV, Mironov AN, Moskalev LI (2005). Deep-sea and shallow-water hydrothermal vent communities: Two different phenomena?. Chem. Geol..

[18] Kongsrud JA, Eilertsen MH, Alvestad T, Kongshavn K, Rapp HT (2017). New species of Ampharetidae (Annelida: Polychaeta) from the Arctic Loki Castle vent field. Deep Sea Res. Part II Top. Stud. Oceanogr..

[19] Kongsrud JA, Rapp HT (2012). *Nicomache (Loxochona) lokii* sp. nov. (Annelida: Polychaeta: Maldanidae) from the Loki’s Castle vent field: An important structure builder in an Arctic vent system. Polar Biol..

[20] Tandberg AH (2012). *Exitomelita sigynae* gen. et sp. nov.: a new amphipod from the Arctic Loki Castle vent field with potential gill ectosymbionts. Polar Biol..

[21] Tandberg AHS, Vader W, Olsen BR, Rapp HT (2018). *Monoculodes bousfieldi* sp. n. from the Arctic hydrothermal vent Loki’s Castle. Mar. Biodiv..

[22] Moalic Y (2012). Biogeography revisited with network theory: Retracing the history of hydrothermal vent communities. Syst. Biol..

[23] Ramirez-Llodra E (2022). Hot vents beneath an icy ocean: The Aurora Vent Field, Gakkel Ridge, revealed. Oceanography.

[24] Ministry of Petroleum and Energy. Mineralverksemd på norsk kontinentalsokkel—opning av areal og strategi for forvaltning av ressursane. *Regjeringa.no* https://www.regjeringen.no/no/dokumenter/meld.-st.-25-20222023/id2985856/ (2023).

[25] Meyer HK (2023). Beyond the tip of the seamount: Distinct megabenthic communities found beyond the charismatic summit sponge ground on an arctic seamount (Schulz Bank, Arctic Mid-Ocean Ridge). Deep Sea Res. Part II Top. Stud. Oceanogr..

[26] Ramirez-Llodra E (2020). Benthic communities on the Mohn’s treasure mound: Implications for management of seabed mining in the Arctic Mid-Ocean Ridge. Front. Mar. Sci..

[27] Washburn TW (2019). Ecological risk assessment for deep-sea mining. Ocean Coast. Manag..

[28] Van Dover CL (2018). Scientific rationale and international obligations for protection of active hydrothermal vent ecosystems from deep-sea mining. Mar. Policy.

[29] Amon DJ (2022). Assessment of scientific gaps related to the effective environmental management of deep-seabed mining. Mar. Policy.

[30] Fautin DG, Barber BR (1999). *Maractis rimicarivora*, a new genus and species of sea anemone (Cnidaria: Anthozoa: Actiniaria: Actinostolidae) from an Atlantic hydrothermal vent. Proc. Biol. Soc. Wash..

[31] Rodríguez E (2008). Morphological phylogeny of the family Actinostolidae (Anthozoa : Actiniaria) with description of a new genus and species of hydrothermal vent sea anemone redefining the family Actinoscyphiidae. Invert. Syst..

[32] Zelnio KA, Rodriguez E, Daly M (2009). Hexacorals (Anthozoa: Actiniaria, Zoanthidea) from hydrothermal vents in the south-western Pacific. Mar. Biol. Res..

[33] Biscoito M (2002). Fishes from the hydrothermal vents and cold seeps—An update. CBM Cah. Biol. Mar..

[34] Eilertsen MH (2018). Genetic connectivity from the Arctic to the Antarctic: *Sclerolinum contortum* and *Nicomache lokii* (Annelida) are both widespread in reducing environments. Sci. Rep..

[35] Georgieva MN (2015). A chemosynthetic weed: the tubeworm *Sclerolinum contortum* is a bipolar, cosmopolitan species. BMC Evol. Biol..

[36] Sen A, Didriksen A, Hourdez S, Svenning MM, Rasmussen TL (2020). Frenulate siboglinids at high Arctic methane seeps and insight into high latitude frenulate distribution. Ecol. Evol..

[37] Buzhinskaja GN, Smirnov RV (2017). A new species of *Raricirrus* (Polychaeta, Ctenodrilidae) from the continental slope of the Laptev Sea near the Gakkel Ridge. Proc. ZIN.

[38] Friele H (1879). Catalog der auf der noprwegischen Nordmeer-expedition bei Spizbergen gefundenen Mollusken. Jahrb. dt. Malakozoolog. Ges..

[39] Warén A (1989). New and little known mollusca from Iceland. Sarsia.

[40] Høisæter T (2009). Distribution of marine, benthic, shell bearing gastropods along the Norwegian coast. Fauna norvegica.

[41] Nekhaev I (2022). *Skenea profunda* (Vetigastropoda: Skeneidae) in the central Arctic Ruthenica. Russ. Malacol. J..

[42] Barnes C, Sweeting CJ, Jennings S, Barry JT, Polunin NVC (2007). Effect of temperature and ration size on carbon and nitrogen stable isotope trophic fractionation. Funct. Ecol..

[43] Hayes JM (2001). Fractionation of carbon and hydrogen isotopes in biosynthetic processes. Rev. Min. Geochem..

[44] Portail M (2016). Food-web complexity in Guaymas Basin hydrothermal vents and cold seeps. PLOS ONE.

[45] Zhulay I, Iken K, Renaud PE, Kosobokova K, Bluhm BA (2023). Reduced efficiency of pelagic–benthic coupling in the Arctic deep sea during lower ice cover. Sci. Rep..

[46] Ziegler AF, Bluhm BA, Renaud PE, Jørgensen LL (2023). Weak seasonality in benthic food web structure within an Arctic inflow shelf region. Progr. Oceanograp..

[47] Bergmann M, Dannheim J, Bauerfeind E, Klages M (2009). Trophic relationships along a bathymetric gradient at the deep-sea observatory HAUSGARTEN. Deep Sea Res. Part I Oceanogr. Res. Pap..

[48] Åström EKL, Bluhm BA, Rasmussen TL (2022). Chemosynthetic and photosynthetic trophic support from cold seeps in Arctic benthic communities. Front. Mar. Sci..

[49] Søreide JE, Hop H, Carroll ML, Falk-Petersen S, Hegseth EN (2006). Seasonal food web structures and sympagic–pelagic coupling in the European Arctic revealed by stable isotopes and a two-source food web model. Progr. Oceanogr..

[50] Young JN, Bruggeman J, Rickaby REM, Erez J, Conte M (2013). Evidence for changes in carbon isotopic fractionation by phytoplankton between 1960 and 2010. Glob. Biogeochem. Cycles.

[51] McCutchan JH, Lewis WM, Kendall C, McGrath CC (2003). Variation in trophic shift for stable isotope ratios of carbon, nitrogen, and sulfur. Oikos.

[52] Bergquist DC (2007). Using stable isotopes and quantitative community characteristics to determine a local hydrothermal vent food web. Mar. Ecol. Prog. Ser..

[53] Kelley, D. S. & Shank, T. M. Hydrothermal systems: A decade of discovery in slow spreading environments. In *Diversity of Hydrothermal Systems on Slow Spreading Ocean Ridges* 369–407 (American Geophysical Union (AGU), 2010). 10.1029/2010GM000945.

[54] Cornelius PFS (1995). North-west European Thecate Hydroids and Their Medusae. Part 1: Introduction, Laodiceidae to Haleciidae. Part 2: Sertulariidae to Campanulariidae. Synop. Br. Fauna (New Series).

[55] Chen C, Hilário A, Rodrigues CF, Ramirez-Llodra E (2022). Integrative taxonomy of a new cocculinid limpet dominating the Aurora Vent Field in the central Arctic ocean. Roy. Soc. Open Sci..

[56] Blake JA, Hilbig B (1990). Polychaeta from the vicinity of deep-sea hydrothermal vents in the eastern Pacific. II. New species and records from the Juan de Fuca and Explorer Ridge systems. Pac. Sci..

[57] Zhang D, Zhou Y, Yen N, Hiley AS, Rouse GW (2023). *Ophryotrocha* (Dorvilleidae, Polychaeta, Annelida) from deep-sea hydrothermal vents, with the description of five new species. Eur. J. Taxon..

[58] Eilertsen MH (2017). Do ampharetids take sedimented steps between vents and seeps? Phylogeny and habitat-use of Ampharetidae (Annelida, Terebelliformia) in chemosynthesis-based ecosystems. BMC Evol. Biol..

[59] Bellan-Santini D (2007). New amphipods of hydrothermal vent environments on the Mid-Atlantic Ridge, Azores Triple junction zone. J. Nat. Hist..

[60] Sasaki, T., Warén, A., Kano, Y., Okutani, T. & Fujikura, K. Gastropods from recent hot vents and cold seeps: Systematics, diversity and life strategies. In *The Vent and Seep Biota: Aspects from Microbes to Ecosystems* (ed. Kiel, S.) 169–254 (Springer Netherlands, 2010). 10.1007/978-90-481-9572-5_7.

[61] Tandberg AHS, Rapp HT, Schander C, Vader W (2013). A new species of *Exitomelita* (Amphipoda: Melitidae) from a deep-water wood fall in the northern Norwegian Sea. J. Nat. Hist..

[62] Criscione F, Ponder WF, Köhler F, Takano T, Kano Y (2017). A molecular phylogeny of Rissoidae (Caenogastropoda: Rissooidea) allows testing the diagnostic utility of morphological traits. Zool. J. Linn. Soc..

[63] Williams ST, Karube S, Ozawa T (2008). Molecular systematics of Vetigastropoda: Trochidae Turbinidae and Trochoidea redefined. Zool. Scr..

[64] Hotaling S, Borowiec ML, Lins LSF, Desvignes T, Kelley JL (2021). The biogeographic history of eelpouts and related fishes: Linking phylogeny, environmental change, and patterns of dispersal in a globally distributed fish group. Mol. Phyl. and Evol..

[65] Govenar, B. Shaping vent and seep communities: Habitat provision and modification by foundation species. In *The Vent and Seep Biota: Aspects from Microbes to Ecosystems* (ed. Kiel, S.) 403–432 (Springer Netherlands, 2010). 10.1007/978-90-481-9572-5_13

[66] Åström EKL, Sen A, Carroll ML, Carroll J (2020). Cold seeps in a warming Arctic: Insights for benthic ecology. Front. Mar. Sci..

[67] Dando, P. R. Biological communities at marine shallow-water vent and seep sites. In *The Vent and Seep Biota: Aspects from Microbes to Ecosystems* (ed. Kiel, S.) 333–378 (Springer Netherlands, 2010).

[68] Portail M (2018). Food-web complexity across hydrothermal vents on the Azores triple junction. Deep Sea Res. Part I Oceanogr. Res. Pap..

[69] Van Dover C (2002). Trophic relationships among invertebrates at the Kairei hydrothermal vent field (Central Indian Ridge). Mar. Biol..

[70] Bourbonnais, A., Lehmann, M. F., Butterfield, D. A. & Juniper, S. K. Subseafloor nitrogen transformations in diffuse hydrothermal vent fluids of the Juan de Fuca Ridge evidenced by the isotopic composition of nitrate and ammonium. *Geochem. Geophys. Geosyst.* **13**, (2012).

[71] Hoch MP, Fogel ML, Kirchman DL (1992). Isotope fractionation associated with ammonium uptake by a marine bacterium. Limnol. Oceanogr..

[72] Sweetman A, Levin L, Rapp H, Schander C (2013). Faunal trophic structure at hydrothermal vents on the southern Mohn’s Ridge. Arctic Ocean. Mar. Ecol. Prog. Ser..

[73] Suh YJ, Kim M-S, Kim S-J, Kim D, Ju S-J (2022). Carbon sources and trophic interactions of vent fauna in the Onnuri Vent Field, Indian Ocean, inferred from stable isotopes. Deep Sea Res. Part I Oceanogr. Res. Pap..

[74] Levin LA (2013). Ecological release and niche partitioning under stress: Lessons from dorvilleid polychaetes in sulfidic sediments at methane seeps. Deep Sea Res. Part II: Top. Stud. Oceanogr..

[75] Thurber AR (2015). Diet-dependent incorporation of biomarkers: implications for food-web studies using stable isotope and fatty acid analyses with special application to chemosynthetic environments. Mar. Ecol..

[76] Toone TA, Washburn TW (2020). Phytodetritus, chemosynthesis, and the dark biosphere: Does depth influence trophic relationships at deep-sea Barbados seeps. Deep Sea Res. Part I Oceanogr. Res. Pap..

[77] Jumars PA, Dorgan KM, Lindsay SM (2015). Diet of worms emended: an update of polychaete feeding guilds. Ann. Rev. Mar. Sci..

[78] Rohlfer EK, Scheer SL, Bergmann M, Sweetman AK, Hoving HJT (2022). Contrasting residence time and scavenging communities of experimental invertebrate food falls in the Arctic deep sea. Deep Sea Res. Part I Oceanogr. Res. Pap..

[79] Stronen AV, Norman AJ, Vander Wal E, Paquet PC (2022). The relevance of genetic structure in ecotype designation and conservation management. Evol. Appl..

[80] Gebruk AV (2003). Methane seep community of the Håkon Mosby mud volcano (the Norwegian Sea): Composition and trophic aspects. Sarsia.

[81] Bell JB (2017). Hydrothermal activity lowers trophic diversity in Antarctic hydrothermal sediments. Biogeosciences.

[82] Lösekann T (2008). Endosymbioses between bacteria and deep-sea siboglinid tubeworms from an Arctic Cold Seep (Haakon Mosby Mud Volcano, Barents Sea). Environ. Microbiol..

[83] Artsdatabanken. Norsk rødliste for arter 2021. https://www.artsdatabanken.no/lister/rodlisteforarter/2021/ (2021).

[84] Panieri G (2024). An Arctic natural oil seep investigated from space to the seafloor. Sci. Total Environ..

[85] Artsdatabanken. Norsk rødliste for naturtyper 2018. https://www.artsdatabanken.no/rodlistefornaturtyper (2018).

[86] Seinhorst JW (1959). A rapid method for the transfer of nematodes from fixative to anhydrous glycerin. Nematologica.

[87] Platt, H. M. & Warwick, R. M. Free Living Marine Nematodes Part II: British Chromadorids. In (eds. Kermack, D. M. & Barnes, R. S. K.) vol. 38 (Published for The Linnean Society of London and The Estuarine and Brackish-Water Sciences Association by E.J. Brill / Dr W. Backhuys, 1988).

[88] Schmidt-Rhaesa, A. in *Handbook of Zoology: Gastrotricha, Cycloneuralia and Gnathifera, Vol. 2: Nematoda*. (Walter De Gruyter, 2014).

[89] Nemys eds. Nemys: World Database of Nematodes. http://www.nemys.ugent.be/ Accessed 01 Nov 2022 (2022).

[90] Huys, R., Gee, J. M., Moore, C. & Hamond, R. *Marine and Brackish Water Harpacticoid Copepods. Part 1: Keys and Notes for Identification of the Species.* (Field Studies Council, Schrewsbury, UK, 1996).

[91] Wells JBJ (2007). An annotated checklist and keys to the species of Copepoda Harpacticoida (Crustacea). Zootaxa.

[92] Sars, G. O. *An Account of the Crustacea of Norway, with Short Descriptions and Figures of All the Species*. 1–1092 (Bergen Museum,1901). 10.5962/bhl.title.1164

[93] Lang K (1948). Monographie der Harpacticiden.

[94] Sarakinos HC, Johnson ML, Zanden MJV (2002). A synthesis of tissue-preservation effects on carbon and nitrogen stable isotope signatures. Can. J. Zool..

