# Supplementary material for: Diversity, habitat endemicity and trophic ecology of the fauna of Loki’s Castle vent field on the Arctic Mid-Ocean Ridge
Source: Sci Rep. 2024 Jan 2;14:103. doi: 10.1038/s41598-023-46434-z (PMC10761849; doi:10.1038/s41598-023-46434-z)
Supplement: Supplementary file 1 — Supplementary Information 1. [file 41598_2023_46434_MOESM1_ESM.docx]

Supplementary material for “Diversity, habitat endemicity and trophic ecology of the fauna of Loki´s Castle vent field on the Arctic Mid-Ocean Ridge” Eilertsen, M.H., Kongsrud, J.A., Tandberg, A.H., Alvestad, T., Budaeva, N., Martell, L., Ramalho, S.P., Falkenhaug, T., Huys, R., Oug, E., Bakken, T., Høisæter, T., Rauch, C., Carvalho, F.C., Savchenko, A.S., Ulvatn, T., Kongshavn, K., Berntsen, C.M., Olsen, B.R., Pedersen, R.B. Scientific Reports.

# Supplementary Notes

## Taxonomy

### Porifera

The only sponge recorded from the active areas of the LCVF is one specimen of the carnivorous sponge *Cladorhiza gelida*, which was sampled from the Barite Field.

### Cnidaria

#### Hydrozoa

Hydrozoans are not common in the LCVF, but a colony of *Hydrallmania falcata* was collected from the vent field in 2015. While the exact collection point for this specimen is not available – and its microhabitat is thus not known – this record is remarkable as it constitutes the only species shared between the LCVF and vents on the Mid-Atlantic Ridge. *Hydrallmania falcata* is a common species in the northern Atlantic Ocean, where it is often found at depths of 100 m or less (Cornelius, 1995), but it has also been recorded from the Lucky Strike hydrothermal vent on the Mid-Atlantic Ridge (Kelley and Shank, 2010). The specimen from LCVF is morphologically indistinguishable from colonies collected in shallower waters (Cornelius, 1995), but a comprehensive analysis integrated with molecular data is required to evaluate the status of any deep-water vent-associated population. All attempts at barcoding the specimen from LCVF have so far been unsuccessful.

#### Actiniaria

Five different species of sea anemones were found at the LCVF, both on hard and soft substrates. These species belong to the actiniarian families Actinostolidae, Hormathiidae, and Kadosactinidae, all of which are known for including taxa associated with deep sea habitats and hydrothermal vents (Fautin and Barber, 1999; Rodríguez et al., 2008; Zelnio et al., 2009). In particular, hormathiid anemones have been sampled in relatively high numbers at LCVF, but which microhabitat these samples were from is uncertain. The different species of kadosactinids occurred at the base of chimneys and in the Oasis diffuse venting area. The Arctic actinostolid species *Anthosactis janmayeni* known from shelf depths in the Arctic Ocean (Riemann-Zürneck, 1997) was present at the base of chimneys on the eastern mound of the LCVF. The specific identity of most of the actiniarians found at the LCVF is still to be determined, as several specimens belong to potential new species or are morphologically highly derived individuals. Because of the lack of species level identification, we did not assess habitat specificity for the actiniarians, except *Athosactis janmayeni,* which is part of the background fauna.

#### Staurozoa

Staurozoans have been observed in the Barite field surrounding fluid outlets on the barite chimneys, and these have previously been identified as *Lucernaria bathyphila* (Pedersen et al., 2010), but unfortunately no sampled specimens were available for identification or barcoding for this study. We still included it in the species list since it has been recorded in the literature.

### Nematoda

Nematodes have previously been reported to be abundant in the worm forests in the Barite Field (Kongsrud and Rapp, 2012). The samples used in this study have not been treated in a manner suitable to preserve the meiofaunal community, and thus mainly nematodes in the macrofaunal size fraction (>0.5 mm) have been identified. The macrofaunal nematode communities at LCVF are predominantly composed of a single Leptosomatidae nematode species, *Platonova magna*, originally described and only reported to date in the eastern slope of the Kuril Islands and its adjacent abyssal sites, in the North-eastern Pacific, at water depth 3374–5152 m (Mordukhovich et al., 2019). In addition, rare observations of a species belonging to a commonly found genus in the macrofauna fraction of deep-sea sediments, *Anticoma* sp. was made, but this species is likely not associated with the worm forest but rather from the adjacent sediments. Another rare record belonged to the genus *Halomonhystera*, but further identification was limited due to the preservation method being inadequate form this small sized specimen. This prevented us from confirming if it might be *Halomonhystera disjuncta*, which is highly abundant (accounting for up to 98% of the nematode fauna) at the Haakon Mosby Mud Volcano (HMMV) (Van Gaever et al., 2006). Habitat specificity was not assessed for the two species identified to genus level, while *Platonova magna* is considered part of the background fauna.

### Nemertea

Small, white nemerteans are sometimes found in samples from the Barite Field. These specimens have not been identified further because of lack of taxonomic expertise.

### Mollusca

#### Gastropoda

Gastropods are among the most abundant taxa at Loki´s Castle, both on the black smoker chimneys and in the diffuse venting areas. There are six species recorded, belonging the families Rissoidae, Skeneidae and Buccinidae, and *Xylodiscula* sp. which is part of the “lower Heterobranchia”. *Rissoa griegi* and *Skenea profunda* are the most abundant taxa, and these species are found both in the diffuse venting areas, and on the black smoker chimneys close to focused venting.

##### Rissoidae

Both *Rissoa griegi* and *Pseudosetia semipellucida* (as *Rissoa semipellucida*) was described by Friele (1879) from a piece of sunken wood colonized by wood-boring bivalves at 2437 m depth (1333 fathoms) west of Svalbard. *Rissoa griegi* has been considered a junior synonym of the shallow water species *Pusillina tumidula* (Warén, 1989), but this synonymisation has later been retracted (Høisæter, 2009). While *P. semipellucida* has been recorded from within the vent field, this species is very rare compared to the extremely abundant *R. griegi*. *P. semipellucida* is known from non-vent deep-sea areas and has also been found in samples from inactive areas the vicinity of Loki’s Castle, and is thus part of the background fauna. The taxonomy of Rissoidae has been largely based on shell characteristics, and a recent molecular phylogeny demonstrated that several of the genera in the family are not monophyletic (Criscione et al., 2017). *Rissoa griegi* has been called *Pseudosetia griegi* in previous publications (Sweetman et al., 2013), but preliminary sequence analyses using the BOLD database shows that *R. griegi* and *Pseudosetia semipellucida* do not cluster together with *Pseudosetia turgida* (type species of *Pseudosetia*) or other *Rissoa* spp. Phylogenetic analyses including both mitochondrial and nuclear markers are needed to resolve the generic placement of these two species.

##### Skeneidae

Two species of *Skenea* have been recorded from LCVF, *Skenea profunda* and *Skenea turgida*. *Skenea profunda* was described from the same piece of wood as *Rissoa griegi* and P*. semipellucida* (Friele, 1879), and has more recently been recorded from the central Arctic Ocean (Nekhaev, 2022). The locality where it was collected in the Arctic Ocean was inferred to have had sunken wood present because in addition to *Skenea profunda*, the wood fall specialist gastropod *Leptogyra bujnitzkii* was collected from the same station (Nekhaev, 2022). The present records from the LCVF demonstrates that *Skenea profunda* is also able to inhabit hydrothermal vents. While *Skenea profunda* is very abundant at LCVF, only a few individuals of *Skenea turgida* has only been collected, and this species belongs to the background fauna.

##### Buccinidae

The buccinid species commonly found in the LCVF has been identified as *Mohnia mohni* (Friele, 1877), which has also been recorded from shallow hydrothermal vents in the Jan Mayen vent field (Schander et al., 2010). However, this is a common species in the deep Norwegian Seas, and thus considered part of the background fauna (Høisæter, 2009).

##### “Lower heterobranchia”

A single individual of *Xylodiscula* sp. has been collected at LCVF. This genus is known from hydrothermal vents on the Mid-Atlantic Ridge, wood-falls and organically enriched coastal areas (Waren and Bouchet, 1993; Høisæter and Johannessen, 2001). Because only a single individual has been collected, the destructive sampling necessary for more thorough studies of morphology and DNA has not been undertaken yet, and the specimen is thus only identified to genus level and habitat selectivity is not assessed.

### Solenogastres

A single individual of Solenogastres was collected from the mounds, but this individual has not been identified further.

### Arthropoda

Pycnogonida

Pycnogonids are not very abundant at LCVF, and only one species - *Ascorhynchus abyssi* Sars, 1877 - has been identified from within the vent field. This species was originally described from deep (1081-1539 m), cold-water localities (Sars, 1877), and has also been recorded from deep-water localities (2200-2600 m) off the coast of northern Norway by the MAREANO programme (Ringvold et al., 2015).

#### Acari

Small marine mites in the family Halacaridae are quite common in samples from the Barite Field and on the hydrothermal mounds. These specimens have not been identified to a lower taxonomic level because of lack of taxonomic expertise.

#### Copepoda

Both calanoid, harpacticoid and cyclopoid copepods have been sampled from LCVF. The six calanoid species identified (*Calanus* *finmarchicus, C. glacialis, C. hyperboreus*, *Aetideopsis rostrata*, *Paraeuchaeta* sp*.*) are well known pelagic species from the region (Wiborg, 1955; Grice and Hulsemann, 1965), and it is possible that some of the samples could have been contaminated by water from higher up in the water column during the ascent of the ROV. However, some of the samples that recovered calanoid copepods are from bladecores, which are kept closed until they arrive on deck. We have also observed calanoid copepods swarming around baited traps on the seafloor in the area nearby LCVF (Eilertsen pers. obs.) which supports that there are calanoid copepods near the seafloor. A cyclopoid copepod of the genus *Cyclopina,* was collected from the Barite Field, and a harpacticoid identified as *Ameira* sp. The specimens identified to *Cyclopina* sp. are believed to be new species to science, supported by morphological characters (R. Huys pers com). Formal species description of the new species has not yet been made and will require further work.

#### Amphipoda

Amphipods are an abundant and diverse group at the LCVF. Two amphipod species have been described previously from the LCVF (Tandberg et al., 2012, 2018), and more await formal description that will follow in separate papers.

##### Calliopiidae

Calliopiids are the most diverse family of amphipods at the LCVF, with potential new species awaiting formal description. The large (up to 30 mm long) and relatively well-known species *Cleippides quadricuspis* Heller, 1875 and *Halirages qvadridentatus* G.O. Sars, 1877 are both found in the vent field, including at the chimney base. The smaller (half size of the two previous species) *Leptamphopus sarsi* Vanhöffen, 1897 is also common in the vent-field, as is an unknown species of *Laothoes*. The family Calliopiidae is in need of revision (d’Udekem d’Acoz, 2012; Ringvold and Tandberg, 2014). All the species described prior to the discovery of LCVF are known from the wider Norwegian Sea, *C. quadridentatus* is regularly found down to 2500 m. The potential new species of calliopiids will, if new, be known only from the LCVF.

##### Hyperiidae

Hyperiids are generally considered to be pelagic amphipods, this is also true for *Themisto libellula* (Lichtenstein in Mandt, 1822) which has been sampled in the vent field, swimming very near to the bottom surface. Its congener *Themisto abyssorum* (Boeck, 1871) is found pelagically in the plume-fluids from LCVF close to the chimney opening (Olsen et al, 2014). Both *Themisto* species are widely distributed and known from depths down to and possibly deeper than 2500m. They are considered important parts of the pelagic foodweb, and seem to feed in a wide variety of depths, including very close to the seafloor (Olsen et al, 2014).

##### Liljeborgiidae

The only species of Liljeborgiidae present at LCVF is *Liljeborgia charybdis* d’Udekem d’Acoz & Vader, 2009. The type locality for this species is at 1700 m near the wrecked submarine *Komsomoletz* (d’Udekem d’Acoz and Vader, 2009), with other suggested findings at West Greenland (1100 m) and southwest of Iceland (1760 m, material from the Ingolf expedition), and presently at Oasis. This is by far the deepest registration of this species, already separated from its other closely related “*Liljeborgia fissicornis-*group" by its deep habitat.

##### Melitidae

The Melitidae are represented by *Exitomelita sigynae* Tandberg, Rapp, Schander, Vader, Sweetman & Berge, 2012, which has its type-locality at LCVF. *E. sigynae* has two morphotypes of chemosynthetic bacteria (methane and sulfid-oxidising) embedded in the outer cuticulum of its gills. All specimens examined have empty guts, even when its mouthparts are highly developed and specialised. *Exitomelita sigynae* is found on the chimneys, in the Barite Field among the tubeworm forest and at Oasis. It is considered a vent specialist and is classified as vulnerable (VU) in the Norwegian Red List for Species (Norwegian Biodiversity Information Centre, 2021).

##### Oedicerotidae

Two species of Oedicerotidae have been identified at LCVF, *Paroediceros lynceus* (M Sars, 1858) and *Monoculodes bousfieldi* Tandberg, Olsen, Vader & Rapp, 2018. While *P. lynceus* is widely known from northern seas, *Monoculodes bousfieldi* is described with type locality and only known distribution at LCVF. It is considered a vent specialist and is evaluated to be vulnerable (VU) in the Norwegian Red List for Species (Norwegian Biodiversity Information Centre, 2021). Only a few species of *Paraoediceros lynceus* was collected, and after sampling for stable isotope analysis and morphological studies, no individuals were available for deposition in the museum collections.

##### Sebidae

Sebidae is represented by *Seba armata* (Chevreux, 1889). This is a species originally described from the Azores (from the expedition *Hirondelle*) at 1287m. The genus is widely distributed, but its species are never found in great abundance.

##### Isopoda

Two small isopod specimens have been collected from the LCVF, but these have not been identified further.

### Annelida

The annelid fauna at Loki’s Castle consists of several species new to science. The ecology and taxonomic status of these species is described briefly below, but formal species descriptions will be published in separate papers.

##### Ampharetidae

There are two species of Ampharetidae described from LCVF; *Paramytha schanderi* and *Pavelius smileyi* (Kongsrud et al., 2017). In addition, a new species in the genus *Anobothrus* has been found that is not yet described. *Anobothrus* sp. nov. is only represented at LCVF by a single specimen collected in 2015 from the worm forest in the Barite Field, but this is a large (approximately 4 cm), well preserved specimen believed to constitute a new species supported by morphological characters and DNA barcodes (Kongsrud et al. in prep). *Anobothrus* sp. nov. is considered to be a vent specialist.

##### Capitellidae

Two species of capitellids in the genus *Notomastus* have been recorded from the LCVF. The first species (*Notomastus* sp. nov. 1) is commonly found in the worm forests in the diffuse venting areas. This species is new to science and is distinguished from other species of *Notomastus* in the region by several morphological characters, the most striking being that it has a more slender appearance. *Notomastus* sp. nov. 1 is considered to be a vent specialist. A second species of *Notomastus* has been found in one sample from the Barite Field, but only three individuals were collected. These specimens of *Notomastus* sp. nov. 2 from LCVF are morphologically most similar to *Notomastus latericus*, which has a type locality in shallow waters off northern Norway (Sars, 1851), but it has a very broad distribution (García-Garza et al., 2019) and might constitute a species complex. *Notomastus* sp. nov. 2 is considered to belong to the background fauna.

##### Cirratulidae

There are two species of Cirratulidae collected from Loki’s Castle, found in the worm forests in the diffuse venting areas. The first species belongs to the genus *Caulleriella* and is a new species to science (Grosse et al. in prep). In addition to being a common occurrence in the Barite Field of LCFV, *Caulleriella* sp. nov. has also been collected from a deep-sea area south of Jan Mayen which is not known to have any hydrothermal activity (Grosse et al. in prep). Because of these records from a non-chemosynthesis hosting environment, this species is considered part of the background fauna. The second species of Cirratulidae from LCVF is *Raricirrus arcticus*, which was described from near the Gakkel Ridge in the Laptev Sea (Buzhinskaja and Smirnov, 2017). In the Laptev Sea, *Raricirrus arcticus* was collected at 2023 m depth along with fossilized shells of *Archivesica* sp., bivalves in the family Vesicomyidae which are well known inhabitants of hydrothermal vents and cold seeps (Sirenko et al., 2004; Buzhinskaja and Smirnov, 2017). Although the bivalve shells were fossilised, Sirenko et al. (2004) noted several characteristics of this site, including sulphidic sediments, which indicates that it might be an active seep site. Therefore, we tentatively consider *R. arcticus* as a CBE-specialist.

##### Dorvilleidae

Dorvilleid worms in the genus *Ophryotrocha* are abundant at Loki’s Castle in sulphidic sediments and represents at least one species new to science. The sampled individuals have a similar morphology, but DNA barcoding indicates cryptic diversity (Oug et al. in prep). Therefore, we here tentatively consider there to be two new species of *Ophryotrocha* from LCVF. These species are currently not known from anywhere else and is thus considered to be vent specialists.

##### Lumbrineridae

One lumbrinerid species that is new to science has been found at Loki’s Castle. The species has also been collected from the Hausgarten observatory (Oug pers. obs.), and from an RP-sled collected in 2009 in the vicinity of Loki´s Castle, but outside the active area. It is thus considered part of the background fauna. The species belongs in a new genus which encompasses four other species that presently are recorded from the deep waters (< 1200 m depth) from north of Iceland, the vicinity of Jan Mayen, the AMOR ridge, and west of Spitsbergen. One of the other new species seems restricted to the cold seep at the Haakon Mosby Mud Volcano (HMMV). The new genus and species are supported by recent DNA sequencing. The five species are morphologically similar, but species-specific characters have between detected in ongoing taxonomic studies (Oug et al., in prep).

##### Maldanidae

The maldanid *Nicomache lokii* Kongsrud & Rapp, 2012 was originally described from LCVF, and has later been recorded from HMMV, Barbados cold seeps and hydrothermal vents in the Southern Ocean (Kongsrud and Rapp, 2012; Eilertsen et al., 2018). At LCVF, *N. lokii* is found both on the base of black smoker chimneys and in the diffuse venting areas, but the species is most abundant in the latter area where it is believed to be important for establishing the worm forest structure together with *S. contortum* (Kongsrud and Rapp, 2012).

##### Orbiniidae

One species of the genus *Orbiniella* has been recorded from Loki´s Castle, which belongs to a species new to science that has also been found in deep waters in the Iceland Sea and Norwegian Sea, and in inactive areas in the vicinity of Loki’s Castle (Meca et al. in press.) and is thus part of the background fauna.

##### Serpulidae

Serpulid worms are very abundant on hard surfaces in active venting areas (around the base of chimneys and in diffuse venting areas, see Figure 2c), but few samples have been collected of good quality. Most specimens have damages such as lacking tentacular crowns or fragmented radioles. They have 7 chaetigerous segments with uncini in 6, and in more complete specimens a globular membranous operculum is observed. Based on these characteristics, specimens are similar to *Protis arctica* (Hansen, 1879; Rzhavsky et al., 2014), but we are reluctant to identify these specimens with accuracy due to conditions of most specimens. *Protis arctica* was originally found in sediments with foraminiferans (Hansen, 1879), and has been reported as widespread in deep-sea habitats with cold water (Rzhavsky et al. 2014). A vent endemic species of the same genus, *Protis hydrothermica*, is known from Pacific hydrothermal vents (Hove and Ztbrowius, 1986), however this species is much larger than the specimens of *Protis* cf. *arctica* from LCVF (tubes of up to 70 mm in length). Because of the uncertainty in the identification of *Protis* cf. *arctica*, habitat specificity is not assessed.

##### Siboglinidae

Two species of siboglinids have been recorded from LCVF – the monoliferan *Sclerolinum contortum* Smirnov, 2000 and the frenulate *Oligobrachia* sp. “Vestnesa” (Sen et al., 2020). *Sclerolinum contortum* is the dominant tubeworm species at LCVF and is well known from this locality as well as cold seeps in the area (Smirnov, 2000; Eilertsen et al., 2018). It has also been recorded from a cold seep in the Gulf of Mexico and sedimented hydrothermal vents in the Southern Ocean, which gives it a very wide geographic distribution (Eilertsen et al., 2018). In 2022, some specimens of *Oligobrachia* sp. “Vestnesa” were collected from the Oasis area, a undescribed frenulate species only previously recorded from the Vestnesa cold seep (Sen et al., 2020). The tubes of *Oligobrachia* sp. “Vestnesa” were found intertwined with *Sclerolinum contortum* tubes, but could be distinguished by being straighter. The identity of the species is supported by DNA barcodes of the COI gene. At the collection site, *Sclerolinum contortum* outnumbered *Oligobrachia* sp. “Vestnesa” by at least an order of magnitude, but a thorough quantification of their relative densities was not possible due to difficulties with telling the worms apart without extracting them from the tube.

##### Sigalionidae

Some polychaetes in the genus *Pholoe* has been collected from the Barite Field, and these are considered to belong to the background fauna.

##### Terebellidae

Two species of Terebellidae have been found at Loki’s Castle, the first species has been identified as a new species of *Phisidia*, which is quite common both in the Barite Field and on the mounds. This species has so far only been recorded from LCVF and is considered a vent specialist. The other species is *Neoamphitrite groenlandica* sensu Holte (1986), which was collected from the Oasis. *N. groenlandica* is a deep-sea species described from Greenland and thus belongs to the background fauna.

### Cordata

Zoarcidae There are two species of zoarcid fish collected within the active areas of the vent field. The first one is *Lycodes paamiuti* Møller, 2001, which is only represented by one sampled specimen. The second species is a still unidentified species of Zoarcidae, which has been collected several times. These small-sized zoarcids are very abundant in the vent field and are often observed within the worm forests in the diffuse-venting areas (Figure 2f). The high abundance and close association with active areas indicates that this could be a vent-endemic species, but because of the unresolved taxonomic status the habitat endemism is not assessed here.

## Supplementary Methods

### Overview map R-script

install.packages("ggOceanMaps")

install.packages("devtools")

devtools::install_github("MikkoVihtakari/ggOceanMapsData") # required by ggOceanMaps

devtools::install_github("MikkoVihtakari/ggOceanMaps")

install.packages("tidyverse")

install.packages("ggnewscale")

install.packages("ggrepel")

library(ggOceanMapsData)

library(ggOceanMaps)

library(tidyverse)

library(ggrepel)

library(ggplot2)

library(ggnewscale)

library(ggspatial)

dtp<- data.frame(lon = c(-20, -20, 15, 15), lat = c(60, 85, 85, 60))

dt_loki <- tibble(lon = c(7.01, 8.16, 14.73,-5.77,-6.25),

lat = c(78.72, 73.56, 72, 71.28, 82.9),

st_id = c('Vestnesa', 'Lokis Castle', 'Håkon Mosby Mud Vulcano', 'JMVF', 'Aurora'),

type = c('Seep', 'Vent', 'Seep', 'Vent', 'Vent'))

basemap(data = dtp, bathymetry = TRUE,

) +

new_scale_fill() +

geom_spatial_point(data = dt_loki, aes(x = lon, y = lat, shape= type, fill=type), color = "black", size = 2, show.legend=TRUE) +

scale_shape_manual(values = c(Seep =21,Vent = 22)) +

scale_fill_manual(values=c(Seep ="#fca503",Vent = "#fc0303")) +

labs(x = "Longitude", y = "Latitude",shape= "Habitat",fill="Habitat") +

ggspatial::annotation_scale()

### Isotope plot R-script

library(ggplot2)

library (tidyverse)

#Ordering species by higher taxon

Isotopes_data_ready$Species <- factor(Isotopes_data_ready$Species, levels = c("Lumbrineridae_gen_et_sp_nov", "Caulleriella_sp_nov", "Nicomache_lokii", "Notomastus_sp_nov", "Ophryotrocha_spp_nov", "Orbiniella_sp_nov", "Paramytha_schanderi", "Phisidia_sp_nov", "Raricirrus_arcticus", "Sclerolinum_contortum", "Calliopiidae_indet", "Exitomelita_sigynae", "Laothoes_sp", "Monoculodes_bousfieldi", "Paroediceros_lynceus", "Seba_armata", "Mohnia_mohni_cluster_A", "Mohnia_mohni_cluster_B", "Rissoa_griegi", "Bythocaris_leucopis", "Actinaria", "Zoarcidae_indet", "Nematoda", "Bacterial_mat"))

#Plotting all specimens as C vs N

first.plot <- ggplot(data = Isotopes_data_ready,

mapping = aes(x = Carbon,

y = Nitrogen)) +

geom_point(aes(shape = Species, color = Species), size = 4) +

scale_x_continuous(breaks = seq(-30, -20, by = 10),

minor_breaks = seq(-40, -10, 1)) +

scale_y_continuous(breaks = seq(-10, 10, by = 10),

minor_breaks = seq(-16, 16, 1)) +

theme_minimal() +

scale_shape_manual(values=c(15,15,15,15,15, 15, 15, 15, 15, 15, 16, 16, 16, 16, 16, 16,17, 17, 17, 13, 8, 6, 5, 3))+

scale_color_manual(values=c("#000000", "#E69F00", "#99ccff", "#009E73", "#F0E442", "#0072B2", "#D55E00", "#CC79A7", "#999999", "#CCFFCC", "#FFCCFF", "#90FFFF", "#663300", "#FFFFCC", "#990099","#999900", "#990000", "#990000", "#006699", "#000000", "#000000", "#000000","#000000","#000000"))+

ylab(expression(paste(delta^{15}, "N (\u2030)"))) +

xlab(expression(paste(delta^{13}, "C (\u2030)"))) +

theme(text = element_text(size=12))

print(first.plot)

# References

Buzhinskaja, G. N., and Smirnov, R. V. (2017). A new species of *Raricirrus* (Polychaeta, Ctenodrilidae) from the continental slope of the Laptev Sea near the Gakkel Ridge. *Proceedings ZIN* 321, 425–432. doi: 10.31610/trudyzin/2017.321.4.425.

Cornelius, P. F. S. (1995). North-west European Thecate Hydroids and Their Medusae. Part 1: Introduction, Laodiceidae to Haleciidae. Part 2: Sertulariidae to Campanulariidae. *Synopses of the British Fauna (New Series)* 50, 1–386.

Criscione, F., Ponder, W. F., Köhler, F., Takano, T., and Kano, Y. (2017). A molecular phylogeny of Rissoidae (Caenogastropoda: Rissooidea) allows testing the diagnostic utility of morphological traits. *Zool J Linn Soc* 179, 23–40. doi: 10.1111/zoj.12447.

d’Udekem d’Acoz, C. (2012). On the genus *Halirages* (Crustacea, Amphipoda), with the description of two new species from Scandinavia and Arctic Europe. *European Journal of Taxonomy*. doi: 10.5852/ejt.2012.7.

d’Udekem d’Acoz, C., and Vader, W. (2009). On *Liljeborgia fissicornis* (M. Sars, 1858) and three related new species from Scandinavia, with a hypothesis on the origin of the group fissicornis. *Journal of Natural History* 43, 2087–2139. doi: 10.1080/00222930903094647.

Eilertsen, M. H., Georgieva, M. N., Kongsrud, J. A., Linse, K., Wiklund, H., Glover, A. G., et al. (2018). Genetic connectivity from the Arctic to the Antarctic: *Sclerolinum contortum* and *Nicomache lokii* (Annelida) are both widespread in reducing environments. *Scientific Reports* 8, 4810. doi: 10.1038/s41598-018-23076-0.

Fautin, D. G., and Barber, B. R. (1999). *Maractis rimicarivora*, a new genus and species of sea anemone (Cnidaria: Anthozoa: Actiniaria: Actinostolidae) from an Atlantic hydrothermal vent. *Proceedings of the Biological Society of Washington* 112, 624–631.

Friele, H. (1877). Preliminary report on the Mollusca from the Norwegian North Atlantic Expedition in 1876. *Nyt Magazin for Naturvidenskaberne* 23, 1–10.

Friele, H. (1879). “Catalog der auf der noprwegischen Nordmeer-expedition bei Spizbergen gefundenen Mollusken,” in *Jahrbücher der Deutschen Malakozoologischen Gesellschaft* (Frankfurt a. M: Verlag von Johannes Alt), 264–286. Available at: https://www.biodiversitylibrary.org/item/55057.

García-Garza, M. E., León-González, J. A. D., and Tovar-Hernández, M. A. (2019). Catalogue of *Notomastus* M. Sars, 1851 (Annelida, Capitellidae) and the description of a new species from the Gulf of California. *Zootaxa* 4577, 249–273. doi: 10.11646/zootaxa.4577.2.2.

Grice, G. D., and Hulsemann, K. (1965). Abundance, vertical distribution and taxonomy of calanoid copepods at selected stations in the northeast Atlantic. *Proceedings of the Zoological Society of London* 146, 213–262. doi: 10.1111/j.1469-7998.1965.tb05210.x.

Hansen, G. A. (1879). Annelider fra den norske Nordhavsexpedition i 1876. *Nyt Magazin for Naturvidenskaberne, Christiania.* 24, 1–17.

Høisæter, T. (2009). Distribution of marine, benthic, shell bearing gastropods along the Norwegian coast. *Fauna norvegica* 28, 5–106. doi: 10.5324/fn.v28i0.563.

Høisæter, T., and Johannessen, P. J. (2001). *Xylodiscula planata* sp. nov., a “lower” heterobranch gastropod from Norwegian waters. *Sarsia* 86, 325–332. doi: 10.1080/00364827.2001.10425522.

Hove, H. A., and Ztbrowius, H. (1986). *Laminatubus alvini* gen. et sp. n. and *Protis hydrothermica* sp. n. (Polychaeta, Serpulidae) from the bathyal hydrothermal vent communities in the eastern Pacific. *Zoologica Scripta* 15, 21–31. doi: 10.1111/j.1463-6409.1986.tb00205.x.

Kelley, D. S., and Shank, T. M. (2010). “Hydrothermal Systems: a Decade of Discovery in Slow Spreading Environments,” in *Diversity of Hydrothermal Systems on Slow Spreading Ocean Ridges* (American Geophysical Union (AGU)), 369–407. doi: 10.1029/2010GM000945.

Kongsrud, J. A., Eilertsen, M. H., Alvestad, T., Kongshavn, K., and Rapp, H. T. (2017). New species of Ampharetidae (Annelida: Polychaeta) from the Arctic Loki Castle vent field. *Deep Sea Research Part II: Topical Studies in Oceanography* 137, 232–245. doi: 10.1016/j.dsr2.2016.08.015.

Kongsrud, J. A., and Rapp, H. T. (2012). *Nicomache* (*Loxochona*) *lokii* sp. nov. (Annelida: Polychaeta: Maldanidae) from the Loki’s Castle vent field: an important structure builder in an Arctic vent system. *Polar Biol* 35, 161–170. doi: 10.1007/s00300-011-1048-4.

Mordukhovich, V. V., Semenchenko, A. A., Fadeeva, N. P., and Zograf, J. K. (2019). One new genus and two new free-living deep-sea nematode species with discussion of phylogeny of the family Leptosomatidae Filipjev, 1916. *Progress in Oceanography* 178, 102160. doi: 10.1016/j.pocean.2019.102160.

Nekhaev, I. (2022). *Skenea profunda* (Vetigastropoda: Skeneidae) in the central Arctic. *Ruthenica, Russian Malacological Journal* 32, 105–109. doi: 10.35885/ruthenica.2022.32(3).2.

Norwegian Biodiversity Information Centre (2021). Norwegian Red List for Species 2021. Available at: https://artsdatabanken.no/lister/rodlisteforarter/2021 [Accessed October 18, 2022].

Pedersen, R. B., Rapp, H. T., Thorseth, I. H., Lilley, M. D., Barriga, F. J. A. S., Baumberger, T., et al. (2010). Discovery of a black smoker vent field and vent fauna at the Arctic Mid-Ocean Ridge. *Nature Communications* 1, 126. doi: www.doi.org/10.1038/ncomms1124.

Riemann-Zürneck, K. (1997). *Anthosactis janmayeni* Danielssen, 1890, a rare high-arctic sea anemone. *Polar Biol* 17, 487–491. doi: 10.1007/s003000050147.

Ringvold, H., Hassel, A., Bamber, R. N., and Buhl-Mortensen, L. (2015). Distribution of sea spiders (Pycnogonida, Arthropoda) off northern Norway, collected by MAREANO. *null* 11, 62–75. doi: 10.1080/17451000.2014.889308.

Ringvold, H., and Tandberg, A. H. S. (2014). A new deepwater species of Calliopiidae, *Halirages helgae* (Crustacea, Amphipoda), with a synoptic table to Halirages species from the northeast Atlantic. *European Journal of Taxonomy*. doi: 10.5852/ejt.2014.98.

Rodríguez, E., Castorani, C. N., Daly, M., Rodríguez, E., Castorani, C. N., and Daly, M. (2008). Morphological phylogeny of the family Actinostolidae (Anthozoa: Actiniaria) with description of a new genus and species of hydrothermal vent sea anemone redefining the family Actinoscyphiidae. *Invert. Systematics* 22, 439–452. doi: 10.1071/IS07053.

Rzhavsky, A. V., Kupriyanova, E. K., Sikrorski, A. V.-, and Dahle, S. (2014). *Calcareous Tubeworms (Polychaeta, Serpulidae) of the Arctic Ocean*. Moscow: KMK Scientific Press Available at: https://www.nhbs.com/calcareous-tubeworms-polychaeta-serpulidae-of-the-arctic-ocean-book [Accessed January 15, 2023].

Sars, G. O. (1877). Prodromus descriptionis crustaceorum et pycnogonidarum, quae in expeditione norvegica anno 1876, observavit. *Archiv for Mathematik og Naturvidenskab* 2, 337–371.

Sars, M. (1851). Beretning om i Sommeren 1849 Fortagen Zoologisk Reise i Lofoten og Finmarken. *Nyt Magazin Naturvidenskaberne.* 6, 121–211. doi: available online at https://www.biodiversitylibrary.org/page/8152707.

Schander, C., Rapp, H. T., Kongsrud, J. A., Bakken, T., Berge, J., Cochrane, S., et al. (2010). The fauna of hydrothermal vents on the Mohn Ridge (North Atlantic). *Marine Biology Research* 6, 155–171. doi: 10.1080/17451000903147450.

Sen, A., Didriksen, A., Hourdez, S., Svenning, M. M., and Rasmussen, T. L. (2020). Frenulate siboglinids at high Arctic methane seeps and insight into high latitude frenulate distribution. *Ecology and Evolution* 10, 1339–1351. doi: 10.1002/ece3.5988.

Sirenko, B., Denisenko, S., Doibel, C., and Rakhor, A. (2004). Deep Water Communities of the Laptev Sea and Adjacent Parts of the Arctic Ocean. *Issledovaniya Fauny Morei* 84, 28–73.

Smirnov, R. V. (2000). Two new species of Pogonophora from the arctic mud volcano off northwestern Norway. *null* 85, 141–150. doi: 10.1080/00364827.2000.10414563.

Sweetman, A., Levin, L., Rapp, H., and Schander, C. (2013). Faunal trophic structure at hydrothermal vents on the southern Mohn’s Ridge, Arctic Ocean. *Mar Ecol Prog Ser* 473, 115–131. doi: www.doi.org/10.3354/meps10050.

Tandberg, A. H., Rapp, H. T., Schander, C., Vader, W., Sweetman, A. K., and Berge, J. (2012). *Exitomelita sigynae* gen. et sp. nov.: a new amphipod from the Arctic Loki Castle vent field with potential gill ectosymbionts. *Polar Biol* 35, 705–716. doi: 10.1007/s00300-011-1115-x.

Tandberg, A. H. S., Vader, W., Olsen, B. R., and Rapp, H. T. (2018). *Monoculodes bousfieldi* sp. n. from the Arctic hydrothermal vent Loki’s Castle. *Marine Biodiversity* 48, 927–937. doi: 10.1007/s12526-018-0869-6.

Van Gaever, S., Moodley, L., de Beer, D., and Vanreusel, A. (2006). Meiobenthos at the Arctic Håkon Mosby Mud Volcano, with a parental-caring nematode thriving in sulphide-rich sediments. *Mar Ecol Prog Ser* 321, 143–155.

Warén, A. (1989). New and little known mollusca from Iceland. *Sarsia* 74, 1–28. doi: 10.1080/00364827.1989.10413419.

Waren, A., and Bouchet, P. (1993). New records, species, genera, and a new family of gastropods from hydrothermal vents and hydrocarbon seeps*. *Zoologica Scripta* 22, 1–90. doi: 10.1111/j.1463-6409.1993.tb00342.x.

Wiborg, K. F. (1955). Zooplankton in relation to hydrography in the norwegian sea. *Report on Norwegian Fishery and Marinc Investigations* 11. Available at: https://imr.brage.unit.no/imr-xmlui/handle/11250/114534 [Accessed April 12, 2023].

Zelnio, K. A., Rodriguez, E., and Daly, M. (2009). Hexacorals (Anthozoa: Actiniaria, Zoanthidea) from hydrothermal vents in the south-western Pacific. *Marine Biology Research* 5, 547–571. doi: 10.1080/17451000902729662.
